# A multi-centre, open label, randomised, parallel-group, superiority Trial to compare the efficacy of URsodeoxycholic acid with RIFampicin in the management of women with severe early onset Intrahepatic Cholestasis of pregnancy: the TURRIFIC randomised trial

**DOI:** 10.1186/s12884-020-03481-y

**Published:** 2021-01-12

**Authors:** William M. Hague, Leonie Callaway, Jennifer Chambers, Lucy Chappell, Suzette Coat, Jiska de Haan-Jebbink, Marloes Dekker, Peter Dixon, Jodie Dodd, Maria Fuller, Sanne Gordijn, Dorothy Graham, Oskari Heikinheimo, Annemarie Hennessy, Risto Kaaja, Teck Yee Khong, Laura Lampio, Jennie Louise, Angela Makris, Corey Markus, Hanns-Ulrich Marschall, Philippa Middleton, Ben W. Mol, Jonathan Morris, John P. Newnham, Caroline Ovadia, Michael Peek, Antonia Shand, Michael Stark, Jim Thornton, Susanna Timonen, Susan Walker, David Warrilow, Catherine Williamson

**Affiliations:** 1 Robinson Research Institute, The University of Adelaide, 55 King William Road, North Adelaide, 5006 South Australia Australia; 2 Obstetric Medicine, Women’s and Babies’ Division, Women’s and Children’s Hospital, 72 King William Road, North Adelaide, South Australia 5006 Australia; 3 Gynaecology, Oncology & Obstetric Medicine, Royal Brisbane and Women’s Hospital, Herston, 4029 Queensland Australia; 4 The University of Queensland, Brisbane, 4072 Queensland Australia; 5 Women and Children’s Health, King’s College London, St Thomas’ Hospital, Westminster Bridge Road, London, SE1 7EH UK; 6 Dutch Consortium for Healthcare Evaluation in Obstetrics and Gynaecology (NVOG Consortium), Postbus 20075, Utrecht, 3502 LB The Netherlands; 7 SA Pathology, Women’s and Children’s Hospital, 72 King William Road, North Adelaide, 5006 South Australia Australia; 8 University of Western Australia Division of Obstetrics and Gynaecology, King Edward Memorial Hospital, PO Box 134, Subiaco, Perth, 6904 Western Australia Australia; 9 Women’s Hospital, University of Helsinki and Helsinki University Hospital, P.O. Box 140, Haartmaninkatu 2, Helsinki, HUS 00029 Finland; 10 School of Medicine, Campbelltown Campus, University of Western Sydney, Narellan Rd, Campbelltown, 2560 NSW Australia; 11 Department of Obstetrics and Gynaecology, Turku University Hospital (TYKS), PO Box 52, Turku, 20521 Finland; 12 Flinders University International Centre for Point-of-Care Testing, College of Medicine & Public Health, GPO Box 2100, Sturt Road, Bedford Park, South Australia 5042 Australia; 13 Wallenberg Laboratory, Sahlgrenska University Hospital, Gothenburg, SE-413 45 Sweden; 14 SAHMRI Women and Kids, South Australian Health and Medical Research Institute, PO Box 11060, Adelaide, 5001 South Australia Australia; 15 Obstetrics & Gynaecology Monash Health, Monash University, Clayton, 3800 Victoria Australia; 16 Paediatrics and Reproductive Medicine, The University of Sydney, Sydney, 2006 New South Wales Australia; 17 Obstetrics and Gynaecology, Australian National University Medical School, The Canberra Hospital, PO Box 11, Woden, 2606 Australian Capital Territory Australia; 18 Obstetrics and Gynaecology, Nottingham University Hospitals NHS Trust, Queen’s Medical Centre, Derby Rd, Nottingham, NG7 2UH UK; 19 Department of Obstetrics and Gynaecology, University of Melbourne, Mercy Hospital for Women, 163 Studley Road, Heidelberg, 3084 Victoria Australia; 20 Public Health Virology Laboratory, Public and Environmental Health, Forensic and Scientific Services, Health Support Queensland, Department of Health, 39 Kessels Road, Coopers Plains, 4108 Queensland Australia

**Keywords:** Intrahepatic cholestasis of pregnancy, Cholestatic pruritus, Bile acids, Ursodeoxycholic acid, Rifampicin, Maternal and neonatal health outcomes

## Abstract

**Background:**

Severe early onset (less than 34 weeks gestation) intrahepatic cholestasis of pregnancy (ICP) affects 0.1% of pregnant women in Australia and is associated with a 3-fold increased risk of stillbirth, fetal hypoxia and compromise, spontaneous preterm birth, as well as increased frequencies of pre-eclampsia and gestational diabetes. ICP is often familial and overlaps with other cholestatic disorders.

Treatment options for ICP are not well established, although there are limited data to support the use of ursodeoxycholic acid (UDCA) to relieve pruritus, the main symptom. Rifampicin, a widely used antibiotic including in pregnant women, is effective in reducing pruritus in non-pregnancy cholestasis and has been used as a supplement to UDCA in severe ICP. Many women with ICP are electively delivered preterm, although there are no randomised data to support this approach.

**Methods:**

We have initiated an international multicentre randomised clinical trial to compare the clinical efficacy of rifampicin tablets (300 mg bd) with that of UDCA tablets (up to 2000 mg daily) in reducing pruritus in women with ICP, using visual pruritus scores as a measuring tool.

**Discussion:**

Our study will be the first to examine the outcomes of treatment specifically in the severe early onset form of ICP, comparing “standard” UDCA therapy with rifampicin, and so be able to provide for the first-time high-quality evidence for use of rifampicin in severe ICP. It will also allow an assessment of feasibility of a future trial to test whether elective early delivery in severe ICP is beneficial.

**Trial identifiers:**

Australian New Zealand Clinical Trials Registration Number (ANZCTR): 12618000332224p (29/08/2018).

HREC No: HREC/18/WCHN/36.

EudraCT number: 2018–004011-44.

IRAS: 272398.

NHMRC registration: APP1152418 and APP117853.

**Supplementary Information:**

The online version contains supplementary material available at 10.1186/s12884-020-03481-y.

## Background

Intrahepatic cholestasis of pregnancy (ICP) is the most frequent liver disorder specific to pregnancy, with an annual Australian incidence of 0.6–0.8% [1]. Its cardinal features are maternal pruritus and increased serum bile acids (BA), commonly towards the end of the third trimester. The pruritus, though often mild, can occasionally be extremely severe, causing major skin excoriations, profound sleep disturbances and associated psychopathology, with iatrogenic premature delivery for symptom relief. ICP can also cause severe liver dysfunction and jaundice. BA are formed in the liver and secreted in the bile into the intestine, where they emulsify fat in the ingested food and enable its uptake into the liver, where the BA are recycled. In ICP, this cycle of “enterohepatic circulation” is inhibited and serum BA concentrations increase, providing a marker for the disorder [2]. Increased rates of postpartum haemorrhage (PPH) reported in early studies [3] have not been confirmed in more recent larger datasets [4]. Gallstones may be present more often in these women [5]. Women with hepatitis C infection have a higher incidence of ICP [6]. Maternal symptoms typically resolve after birth, but affected women have an increased risk of hepatobiliary disease in later life [6]. There are few data for long term outcome in the children of mothers with ICP [7].

ICP has been associated with increased fetal risks, including spontaneous preterm birth, fetal hypoxia and compromise (including meconium-stained liquor) and unexpected intrauterine fetal death [3]. Higher concentrations of circulating BAs are associated with increased fetal risks, including spontaneous preterm birth, asphyxial events and stillbirth [8, 9]. Early onset (less than 34 weeks gestation) severe (defined as serum BA > 40 μmol/L) ICP is a rare disorder with an Australian incidence of 0.1% per annum [1].

The aetiology of ICP has been linked to increased concentrations of oestradiol, progesterone and, in particular, of progesterone metabolites in pregnancy, which impact the farnesoid X receptor (FXR)-mediated BA homeostasis pathways [10]. Why only some women develop ICP may be attributable to genetic variants of the genes encoding biliary transporters, eg phosphatidyl-choline floppase multidrug resistance protein 3 (MDR3; *ABCB4*), Bile Salt Export Pump (BSEP; *ABCB11*), phosphatidyl serine flippase (*ATP8B1*), the multidrug resistance-associated protein 2 (MRP2; *ABCC2*), and also tight junction protein 2 (TJP2), [11] together with FXR, which is critical in the transcriptional activation of both *ABCB11* [12] and *ABCB4* [13]. Further evaluation of genetic variants that confer ICP susceptibility may allow precision medicine in the identification of women at increased risk of adverse pregnancy outcome, and also those more likely to respond to targeted treatment of ICP with specific drugs.

A number of agents from different pharmacological classes have been used in the management of women with ICP. These include: ursodeoxycholic acid (UDCA), cholestyramine, dexamethasone, s-adenosylmethionine and, most recently, rifampicin. None are based on high level evidence and, as a consequence, there are various concerns over costs, side effects and possible harms. The publication of the PITCHES study, a placebo-controlled RCT of UDCA in 605 women with ICP of any severity recruited between 20 and 40^+ 6^ weeks gestation, has confirmed a small reduction in pruritus for UDCA but no benefit in respect of a composite of adverse perinatal outcomes [14]. Pharmacological treatment of ICP is the subject of a 2020 Systematic Review in the Cochrane Library [15]. Included in this review were 26 randomised controlled trials involving 2007 participants. The trials were mostly at moderate to high risk of bias. Compared with placebo, the pooled results showed a probable small improvement in pruritus score measured on a 100 mm visual analogue scale (VAS) (mean difference (MD) − 7.64 points, 95% confidence interval (CI) − 9.69 to − 5.60 points for UDCA in two trials (715 women) (GRADE moderate certainty). The evidence for fetal compromise and stillbirth were uncertain, due to serious limitations in study design and imprecision (risk ratio (RR) 0.70, 95% CI 0.35 to 1.40; 6 trials, 944 women; RR 0.33, 95% CI 0.08 to 1.37; 6 trials, 955 women; GRADE very low certainty).

Understanding of the mechanism of action of the various drugs is limited. Ursodeoxycholic acid (UDCA) is a naturally occurring BA, which displaces more hydrophobic endogenous bile salts from the BA pool. This action may shield the hepatocyte membrane from the damaging toxicity of bile salts, enhancing BA clearance across the placenta from the fetus [16]. UDCA has also been shown in vitro to protect rat cardiomyocytes from damage by endogenous bile salts [17].

Since the publication of the previous Cochrane review in 2013 [18], there has been increasing interest in the use of rifampicin in treating women with ICP. Rifampicin is a semisynthetic antibiotic with a wide range of antimicrobial activity and is a first-line agent for treatment of tuberculosis, including for treatment of pregnant women [19, 20]. Rifampicin has also been shown to have the capacity to reduce serum BA in the management of cholestasis outside of pregnancy. It is a pregnane X-receptor (PXR) agonist and a potent inducer of key enzymes in the hepatic and intestinal detoxification machinery (such as CYP3A4, CYP2D, UGT1A1, SULT2A1) and export pump MRP2 [21]. A systematic review of pharmacological interventions for pruritus in palliative care showed that, in patients with cholestatic pruritus, data favoured the use of rifampicin, showing a low incidence of adverse events when compared with placebo [22]. There have been no reported trials comparing UDCA and rifampicin in the treatment of pruritus in any clinical scenario. Rifampicin has no known teratogenic effects, although an effect on vitamin K metabolism mandates parenteral neonatal administration of vitamin K [23]. In a questionnaire survey of clinicians, who reported results on 27 women with 28 affected pregnancies identified via the International Obstetric Medicine forum: the clinical case notes of women with ICP treated with UDCA and rifampicin were reviewed, and data regarding maternal and perinatal outcomes were extracted [24]. While serum BA remained high whilst taking UDCA as monotherapy, use of rifampicin led to reductions in serum BA in more than half the women, and to halving of serum BA in more than 33%. No adverse effects were reported with either drug. There were no stillbirths and no postpartum haemorrhage reported, but the numbers were very small.

Data from The Netherlands suggest that rifampicin may be a more appropriate therapy for individuals with *ABCB11* mutations associated with clinical BSEP deficiency and Benign Recurrent Intrahepatic Cholestasis (BRIC) outside of pregnancy [25]. Recent data from France, on the other hand, while supporting the use of UDCA in ICP, did not show any relation of *ABCB4* mutations to treatment with UDCA [26].

Other strategies to improve outcomes for pregnant women have focussed on the timing of birth. A trial involving 62 women with ICP (any gestation or severity) compared early term delivery (induction or delivery started between 37^+ 0^ and 37^+ 6^ weeks gestation) versus expectant management (spontaneous labour awaited until 40 weeks gestation or caesarean section undertaken for normal obstetric indications, usually after 39 weeks gestation). There were no stillbirths or neonatal deaths in either group. No clear differences in caesarean section, passage of meconium-stained liquor or admission to neonatal intensive care unit were observed [27]. Despite the lack of RCT evidence to support benefit for early delivery and potential harms, many centres around the world have adopted a practice of ensuring birth for women by 37 weeks gestation, particularly of women with severe ICP, to avoid the increased risk of stillbirth [28].

We have therefore planned a series of trials to answer some of these important clinical questions, using a similar plan as the protocol for the prospective STRIDER studies [29]. In this initial study, we compare the use of rifampicin vs UDCA in women with severe early onset ICP. The trial has as its primary outcome pruritus, an important clinical endpoint for the women affected, which can be measured easily and effectively with a well-validated visual scale as a continuous variable. It also allows a much smaller sample size in a superiority design than that required to demonstrate differences in perinatal morbidity and mortality. The results of the trial will then be able to be compared, subject to HREC and local governance approval after submission of a separate protocol, data sharing agreements and appropriate de-identification of the data, in a meta-analysis with the results from the PITCHES trial to give a robust estimate of the sample size required to conduct a definitive clinical trial with serious perinatal morbidity and mortality as major outcomes.

Our study will be the first to examine the outcomes of treatment specifically in the severe early onset form of ICP, comparing “standard” UDCA therapy with rifampicin, and so be able to provide for the first-time high-quality evidence for use of rifampicin in severe ICP.

## Methods/design

### Trial hypotheses

The primary trial hypothesis is that rifampicin, compared with UDCA treatment of women with severe early onset ICP, commenced between 14^+ 0^ and 33^+ 6^ weeks of gestation, reduces the degree of pruritus, measured as worst itch in the previous 24 h assessed on a patient-recorded visual analogue scale.

Secondary trial hypotheses include:

- Rifampicin, compared with UDCA, improves short-term outcomes for both mother and infant in severe early onset ICP, including the length of gestation and the incidence of caesarean section and preterm birth.
- Rifampicin, compared with UDCA, improves markers of maternal liver function in severe early onset ICP, including serum BA and serum transaminase concentrations.

### Trial design

TURRIFIC is a multicentre randomised open label controlled study to evaluate whether rifampicin is superior to UDCA in the reduction of pruritus in parallel groups of women with severe ICP presenting between 14^+ 0^ and 33^+ 6^ weeks gestation. Initial random allocation to treatment with rifampicin or UDCA is in a ratio of 1:1.

### Trial setting

The study is being conducted in11 academic hospital centres across Australia and internationally in the UK, Sweden, Finland and The Netherlands. [See list at www.adelaide.edu.au/TURRIFIC].

### Participants

Potentially eligible participants are identified via antenatal clinics, antenatal assessment units and antenatal wards and by review of laboratory BA results.

Women attending for management of severe early onset ICP are invited by the treating physician/obstetrician to consider participation in the study, and if agreeable, provide written informed consent to participate.

### Inclusion criteria

Women are considered eligible for inclusion into the trial with the following criteria:

- Severe ICP (defined as pruritus with increased total serum BA ≥40 μmol/L) confirmed
- Viable pregnancy between 14^+ 0^ and 33^+ 6^ weeks gestation inclusive (as determined by ultrasound pregnancy dating)
- No known lethal fetal anomaly
- Singleton pregnancy
- Obstetric care in a consultant-led unit
- Aged 18 years or over
- Written informed consent has been obtained

Standardisation of serum BA concentration measurements: participating units will have tested a series of serum samples sent out from Adelaide, for comparison and correlation with the Adelaide values, and subsequent adjustment of the results from the local laboratories to enable standardisation of the results across the trial. Similar quality control testing will be carried out every 12 months.

Women who present with non-severe ICP (total serum BA < 40 μmol/L) between 14^+ 0^ and 33^+ 6^ weeks gestation, and who then progress, with or without UDCA treatment, to severe ICP (total serum BA ≥40 μmol/L) prior to 34^+ 0^ weeks gestation, are eligible for entry into the trial, providing they are willing to accept random allocation to treatment (with a wash-out period of any pre-existing UDCA treatment for 4–7 days to enable baseline biochemical data to be obtained, and confirm eligibility only if serum BA were measured ≥40 μmol/L while not taking UDCA), they meet the other inclusion criteria and they do not meet any of the exclusion criteria (below), in which case they will be randomised either to rifampicin therapy or to commence/continue with UDCA therapy.

### Exclusion criteria

A potential participant who meets any of the following criteria will be excluded from participation in this study:

- A decision has already been made for delivery within the next 48 h
- There is allergy to any component of the UDCA or rifampicin tablets
- The woman is taking other medication that has a significant interaction with rifampicin treatment, including saquinavir and ritonavir in combination for treatment of HIV, daclatasvir, simeprevir, sofosbuvir and/or telaprevir for treatment of hepatitis C, isoniazid and/or pyrazinamide for treatment of tuberculosis
- General anaesthesia within the previous 3 months to avoid the risk of inadvertent and unrecognised previous halothane exposure
- There is a multi-fetal gestation
- There is laboratory-confirmed active hepatitis A or hepatitis B, or positive serology for hepatitis C
- There is current pre-eclampsia (ISSHP criteria) [30]
- There is a known active primary hepatic disorder, including α-1-antitrypsin deficiency and autoimmune hepatitis, including primary biliary cholangitis
- The woman is currently taking medication, which causes deranged liver enzyme values
- The woman is unwilling for her baby to have standard vitamin K administration at birth
- The woman has previously participated in TURRIFIC

A woman will not be excluded, and may be randomised, if she is known to have:

- A known genetic disorder associated with cholestasis
- Asymptomatic cholelithiasis (no colicky right upper quadrant pain/jaundice)
- Current gestational diabetes (GDM) (WHO criteria) [31]

## Interventions

The intervention under test is rifampicin (rifampicin) tablets 300 mg twice daily and the comparator treatment is ursodeoxycholic acid (UDCA) tablets up to 2000 mg daily, prescribed as per the usual local protocols.

Women meeting the inclusion criteria and consenting to participation will be randomly allocated to treatment with rifampicin or UDCA at study entry. The duration of treatment will range from 1 day to a maximum of 28 weeks, for a participant randomised at 14 weeks who does not deliver until 42 weeks.

Within the trial, both UDCA and rifampicin are usually dispensed by the local hospital pharmacy.

### Ursodeoxycholic Acid (UDCA)

There are no known drug contraindications to UDCA.

The starting dose of UDCA is 450-1000 mg daily (450 mg or 500 mg tablets as per local pharmacy stock to be taken once daily or twice daily), increased in increments of 500 mg per day every 3–14 days, if there is no biochemical/clinical improvement, up to a maximum of 2000 mg per day [32]. UDCA will be continued until birth and then discontinued, or it may be weaned at the discretion of the treating clinician.

### Possible side-effects

Common side effects include soft loose stools or diarrhoea.

Less common side-effects include urticaria.

### Rifampicin

The dose of rifampicin is 300 mg twice daily. Administration of rifampicin will be withheld if, during treatment, there is a rise of serum ALT above 200 U/L, and the management of such patients discussed with senior clinicians, including CI Hague and/or CI Williamson, to decide about continuation of rifampicin therapy. Rifampicin will be weaned after birth by 150 mg every 3 days.

### Possible side-effects

Commonly, body fluids, including urine, stools, saliva, sputum, sweat and tears, turn red-orange.

Other side effects from rifampicin are not common, but can include headache, muscle pain, bone pain, heartburn, upset stomach, vomiting, stomach cramps, chills, diarrhoea, urticaria, sores on skin or in the mouth, fever, or jaundice.

### Drug interactions

Halothane, listed in the Product Information as an exclusion for rifampicin treatment, is not currently used in the countries where the trial is being conducted. It is possible that immigrant women may have been exposed to halothane before arrival. All women being screened for eligibility will be asked if they have had a general anaesthetic within the previous 3 months and, if so, they will be excluded from the trial to avoid the risk of inadvertent and unrecognised previous halothane exposure. It is most unlikely that women with active or currently treated tuberculosis (TB) will be recruited to the trial, but women taking isoniazid or pyrazinamide will not be considered for inclusion.

Rifampicin can have significant interaction with other drug therapies. In particular, it can interact with hormonal contraceptive agents and appropriate advice will be given in the postpartum period for those women who have been taking rifampicin to take different or additional contraceptive precautions for a month after ceasing rifampicin therapy.

### Current practice

Women who present with mild to moderate ICP remote from term will usually be offered treatment with UDCA, with ongoing surveillance in the obstetric and obstetric medical clinics, and consideration given to delivery between 38 and 40 weeks gestation.

Women who present with, or who progress to, severe ICP will usually be offered treatment with increasing doses of UDCA, with the addition of rifampicin, if considered necessary, to reduce the serum BA concentrations, even though this is not a standard indication. Delivery will often be brought forward to between 36 and 37 weeks gestation, especially if there is subjective reduction in fetal movements.

### Adherence to and returns of study treatment

Treatment adherence will be measured by review of the medication dosing card and participant self-report at each visit. Medication dosing cards will be collected when they are completed and at the end of the study.

There is no compulsion to return any unused medication. However, study staff will offer to dispose safely of any unused medications that the participant wishes to relinquish.

### Concomitant medication

All prescribed and “over the counter” medications deemed necessary by an attending healthcare professional to provide adequate supportive care to the woman are permitted during the trial. All medications taken by the woman, including vitamin K and other supplements, are to be recorded in the participant’s eCRF.

Women taking rifampicin and who are subsequently commenced on medications known to have significant interaction with rifampicin will be withdrawn from the trial.

## Trial outcomes

### Primary outcome

Maternal pruritus score, measured as worst itch in the previous 24 h assessed on a patient-recorded visual analogue scale.

### Secondary outcomes

The secondary short-term maternal outcomes are defined as:

- Serial biochemical indices of disease: serum concentration of bile acids (BA), bilirubin (total), alanine transaminase (ALT), gamma glutamyl transferase (GGT)
- Peak serum concentration (between randomisation and delivery) of BA
- Maximum doses of trial medications required, and days of such medications
- Days from randomisation to birth
- Days to resolution/amelioration of symptoms
- Need for added treatment with UDCA or rifampicin as appropriate after 7 days of the randomly allocated drug therapy
- Need for additional therapy at maximum trial dosage (e.g. antihistamines, cholestyramine, therapeutic plasma exchange/other)
- Incidence of gestational diabetes mellitus (WHO criteria) and its treatment (diet/metformin/insulin), and of gestational hypertension/pre-eclampsia (ISSHP criteria)
- Upper abdominal ultrasound/biliary pathology
- Mode of onset of labour, and gestation at onset
- Timing of steroids (if any) for fetal pulmonary maturation
- Duration of labour
- Presence of meconium in the liquor intrapartum
- Gestation at birth
- Mode of birth, classified as spontaneous vaginal, instrumental vaginal or Caesarean
- Reason for induction or pre-labour Caesarean section
- Estimated blood loss at birth
- Time for resolution of symptoms after birth

The secondary short-term perinatal outcomes are defined as:

- Miscarriage (fetal death before 20^+ 0^ weeks gestation)
- Stillbirth (death before delivery ≥20^+ 0^ weeks gestation or > 400 g if gestation unknown)
- Neonatal death in hospital up to 7 days after birth (excluding death due to congenital anomalies)
- Neonatal unit admissions until infant discharge home from hospital
- Number of nights in each category of care (intensive, high dependency, special, transitional and normal) and total number of nights in hospital
- Birth weight (g), and customised/population birth weight centile (GROW) [33]
- Apgar scores at 1 and 5 min after birth
- Umbilical arterial and venous pH (and base excess) at birth
- Cord blood BA
- Need for supplementary oxygen prior to discharge, and number of days when such oxygen is required
- Need for ventilation support (continuous positive airway pressure - CPAP/high flow/endotracheal ventilation)
- Pneumothorax (confirmed on chest X-ray)
- Need for phototherapy
- Abnormal cerebral ultrasound scan
- Confirmed sepsis (positive blood or cerebrospinal fluid cultures)
- Necrotising enterocolitis (Bell’s stage 2 and 3)
- Seizures (confirmed by EEG or requiring anticonvulsant therapy)
- Encephalopathy grade (worst at any time: mild, moderate, severe)
- Hypoglycaemia (blood glucose < 2.6 mmol/l on two or more occasions)
- Severe hypoglycaemia (blood glucose < 1.8 mmol/l on two or more occasions)
- Other indications and main diagnoses resulting in NNU admission.
- Mode of feeding at discharge from hospital

The primary outcome, maternal pruritus score, will be evaluated at 1 week after trial entry and then on a monthly basis up to 28^+ 0^ weeks gestation and then weekly to delivery.

The timepoints of evaluation of the secondary maternal outcomes are at 1 week post-randomisation visit, at monthly clinic visits at 20, 24 and 28 weeks gestation (if randomised prior to that), at weekly clinic visits after 28 weeks gestation and during admission for birth, and of the secondary neonatal outcomes at delivery and 1 week and 6 weeks after birth.

The following information post enrolment will be captured for health resource use:

- Maternal: total number of nights (antenatal, intrapartum and postnatal) together with level of care including adult ICU.
- Infant: total number of nights for the baby in neonatal unit, together with level of care (e.g. neonatal ICU).

The costs of UDCA and rifampicin, together with the costs of any additional treatment, will be calculated in both the UDCA and rifampicin groups.

## Main trial procedures (see timeline in Table 1)

### Informed consent

Written informed consent will be sought from the woman only after she has been given a full verbal explanation (by a healthcare professional or research midwife/assistant) and written description (via the most recent approved version of the PICF in the local national language). She will be given sufficient time to consider the information, and the opportunity to question the PI or other independent parties to decide whether she will participate in the trial. Women who do not speak English, or the national language if in a non-Australian centre, will only be approached if an interpreter is available.

**Table 1:**
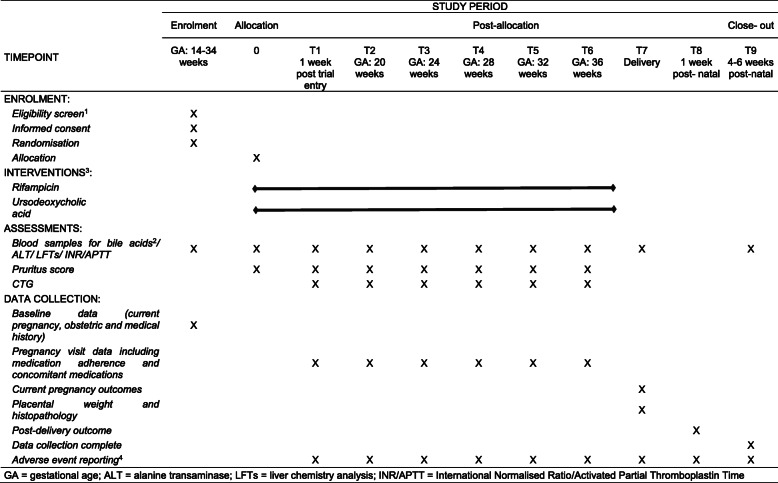
Trial Timeline

*GA* gestational age, *ALT* alanine transaminase, *LFTs* liver chemistry analysis, *INR/APTT* International Normalised Ratio/Activated Partial Thromboplastin Time

Notes to Table 1

^1^All screening assessments are part of normal clinical practice

^2^Bile acid samples need to be drawn shortly before a scheduled dose of UDCA, though fasting is not required. Women previously prescribed UDCA for treatment of mild ICP, and agreeing to randomisation, will have basal assessments performed 4–7 days after ceasing such therapy prior to randomisation into the trial

^3^Study treatment started after randomisation. Dose to be adjusted by PI if indicated by symptoms and/or blood tests taken during normal clinical practice

^4^Adverse events will be recorded from study entry until post-delivery discharge of woman and baby. All SAEs are to be reported to the Coordinating Centre within 24 h of Investigator knowledge

Women eligible for the trial, whether or not they participate, will be asked about their willingness to take part in a subsequent trial of planned delivery of such affected women at 36^+ 0^ weeks gestation compared with expectant management in terms of neonatal outcomes.

Women taking part in the study will be asked about any other drugs that have been prescribed or initiated after randomisation. If such medications include agents known to have a significant interaction with rifampicin, then a woman taking rifampicin will be asked to withdraw from the study, although any data obtained until withdrawal will be included, and any pregnancy outcome data will be recorded (with her consent).

Participating women will also be asked at recruitment about any antibiotics they may have taken in the 3 months prior to recruitment, as well as any progesterone supplements they may have taken.

### Sample size

It is planned to recruit 108 women in total.

### Randomisation

Initial random allocation to treatment with rifampicin or UDCA will be in a ratio of 1:1.

Randomisation is managed via a secure web-based randomisation facility hosted on REDCap at The University of Adelaide, with telephone back-up available.

Stratification is by centre and by severity (serum BA concentration prior to randomisation < 100 μmol/L, ≥100 μmol/L).

The woman is allocated a unique participant identification number on randomisation.

### Baseline assessments

Baseline data, including all demography, itch chart results, serum BA concentrations, other serum analytes, viral serology and coagulation profiles, including prothrombin time and activated partial thromboplastin time (recorded within 7 days prior to randomisation, and 4–7 days after cessation of any previously initiated UDCA therapy), are entered on the web-based data base. The itch chart is a previously validated 100 mm visual analogue score where the participant is asked to score her worst itch in the previous 24 h [27]. All laboratory data are measured in the routine hospital laboratories for each centre.

Other biological samples, including serum, urine, faeces, liquor and placenta, may also be collected (with participant consent) under separate protocols and stored for later assessment.

On completion of these details, the database issues a randomisation notification to the site PI/delegate. A prescription for the allocated study treatment is written by the local PI or their delegate, using a standard clinical prescribing script, and the participant collects the study treatment from the pharmacy.

### Subsequent visits

At the first visit a week after randomisation, and then at least monthly, if randomised prior to 28 weeks gestation, and then weekly thereafter, the woman is asked for a value of the worst itch she felt during the previous 24 h, which is then scored on the itch chart. Serum is drawn and monitored for measurement of BA and liver biochemistry and measurement of prothrombin time and activated partial thromboplastin time (as per usual clinical practice), together with any further biological samples for later assessments (with the woman’s consent) under separate protocols, together with routine cardiotocography and ultrasound monitoring (weekly umbilical artery Doppler, 2-weekly growth parameters). The dose of treatment is altered at the discretion of the responsible clinician.

If maximal doses of primary drug therapy are reached, consideration is given to the addition of, first UDCA or rifampicin respectively, following measurement of any appropriate biochemical and microbiological samples, to enable the relevant comparisons to be drawn, and then other therapy, eg cholestyramine, in addition to the trial therapy.

Women in the study, whether randomised to either UDCA or rifampicin, who develop steatorrhoea, are also to be prescribed vitamin K supplements (10 mg daily) to be taken until delivery.

Women may be prescribed pre-delivery antenatal steroids for enhancing lung maturity at the discretion of the treating clinician. Any such antenatal steroid therapy will be recorded in the eCRF.

The remainder of antenatal care and, in particular, decisions about the mode of birth as well as the administration of steroids for promotion of fetal lung maturity, is left to the discretion of the treating clinician. All neonates, and especially those of women taking rifampicin, are to be given parenteral Vitamin K at birth.

The post-delivery outcome will be completed after delivery and before the woman leaves hospital.

Participants will be asked to attend for follow-up at 6 weeks postpartum, with routine blood samples (including BA measurement as well as OGTT, if appropriate) collected 1–3 days beforehand.

### Biological samples and laboratory tests

Routine biochemical, autoimmune and viral serology, and coagulation testing as per local maternity unit guidelines (commonly serum BA, liver biochemistry, including ALT/GGT/bilirubin, viral serology including hepatitis A, B, C, D, E and G (as per local guidelines), cytomegalovirus (CMV), Epstein-Barr virus (EBV) and herpes simplex (HSV), autoimmune liver serology (mitochondrial, smooth muscle, ANA and LKM antibodies) and INR/APTT).

Serum BA do not need to be collected fasting but should be collected shortly before the next scheduled dose of UDCA, if being taken. Fasting/non-fasting and the length of time since the last dose of UDCA should be noted.

### Withdrawal of participants

It will be made clear to participants that they remain free to withdraw from the clinical trial at any time without the need to provide any reason or explanation; and that this decision will not impact on any aspect of their ongoing clinical care. The investigator will be able to discontinue participants from either of the study drugs in the event that they judge that alternative treatment is required, without the woman being necessarily withdrawn from the study. Participants will not be withdrawn for self-reported low or non-adherence to their study treatment.

Permission will be sought to complete and use data up to the point of withdrawal including both:

- trial specific data
- data collected as per routine clinical practice

Permission will also be sought to ascertain and record subsequent perinatal outcome data.

The reason for withdrawal will be recorded in the eCRF, and, if it was due to an Adverse Event, the investigator will follow up to resolution/stability.

Similarly, it will also be made clear to participants that they remain free to withdraw from any future sub-studies to which they have agreed at any time without the need to provide any reason or explanation; and that such a decision will not impact on any aspect of their ongoing clinical care.

## Statistics

### Rationale for sample size

One hundred eight women are required to have a 90% chance of detecting, as significant at the 5% level, a decrease in maternal pruritus score from -16 mm in the UDCA group to -23 mm in the rifampicin group, (with a standard deviation (SD) of 10 mm across both arms, allowing for a 5% drop out in each arm).

The SD stipulated for the sample size calculation might appear to be too small, on the basis of the values reported in the PITCH study, in which UDCA reduced itching by -16 mm (95% confidence interval -27 mm to -6 mm) [27].. The estimate and 95% Confidence Interval for the difference in pruritus score between the UDCA and the Placebo group was -16 mm (-26.5,  -5.9 mm). This suggests that the standard error is approximately 5.3 mm, and hence the standard deviation of the difference between the groups (given *n* = 55 and *n* = 56) is approximately 28 mm.

The statistical power of the study, however, has been calculated with the intended sample size of 108 (*n* = 54 per group), so as to detect a difference between UDCA and rifampicin if the mean change in pruritus score in the UDCA group is -16 mm (as reported in the PITCH study versus placebo, where change in the placebo group was negligible), while the mean change in pruritus score in the rifampicin group is -30 mm (which has already been stipulated as the minimum change that would be considered clinically meaningful). These calculations were done as the power for a simple comparison of two means, and via simulation to allow for up to five measurements per participant, with correlation between the repeated measures, and show that there is approximately 75% power (with two-sided alpha 0.05) to detect a difference between the groups of this magnitude; i.e., if rifampicin achieves a clinically meaningful reduction in pruritus score while UDCA achieves a reduction of the magnitude reported in PITCH, there is 75% power to detect this.

In the recent PITCHES study, however, the reduction of pruritus score in the UDCA group was much more modest (-5.7 mm) [14]. The CI gives a very similar SD, i.e. 25 mm. This gives much greater power to detect a difference between UDCA and rifampicin: if rifampicin had a change of -23 mm, we have approx. 95% power, and if it had a mean change of -30 mm, > 99% power.

The inclusion of multiple centres will enable maximum recruitment over a limited time scale.

### Data analysis

A detailed Statistical Analysis Plan will be developed by the trial statistician (who is independent of the Sponsor) and agreed by the Trial Management Committee (TMC) before the analysis is undertaken. The analysis and presentation of results will follow the most up-to-date recommendations of the CONSORT group. Analyses will be completed in STATA® version 13.0 or later.

All analyses will follow the intention to treat principle, i.e. data from all randomised woman and babies will be analysed according to the treatment they were allocated to irrespective of the treatment they received or whether they received any treatment at all.

Blinding will not be possible for participants and care providers, because of the open nature of the interventions, nor will the DSMC be blinded to allocation in their assessment of outcomes and safety, but the outcome data will be blinded to the data analysts by concealing the group allocation prior to the analysis.

Demographic and clinical data will be summarised with counts and percentages for categorical variables, means (standard deviations) for normally distributed continuous variables and medians (with interquartile or simple ranges) for other continuous variables.

For the primary outcome, the effectiveness of the interventions will be assessed by calculating the differences in mean pruritus score with 95% confidence intervals (CI), determined with a linear regression model, using Generalised Estimating Equations (GEEs).

All comparative analyses will be performed adjusting for stratification variables and baseline measures of the outcome, where relevant. Binary outcomes will be analysed using log binomial regression models. Results will be presented as adjusted risk ratios plus 95% CIs. If the model is unstable, log Poisson regression models with robust variance estimation will be used [34]. Continuous outcomes will be analysed using linear regression models, and results will be presented as adjusted differences in means (with 95% CIs). Analysis of outcomes that are measured repeatedly over time (severity of pruritus and biochemical markers) will use repeated measures analysis techniques, including GEEs and/or random effects.

### Pre-specified subgroup analysis

Pre-specified subgroup analyses will use the statistical test for interaction and where appropriate, results will be presented as differences in means with 95% CIs, and the number needed to treat to benefit or harm calculated. Pre-specified subgroups will be based on:

- Serum BA at baseline (< 100 μmol/L, ≥100 μmol/L).
- Site

### Dealing with missing data

Missing data as a result of women or babies being lost to follow-up are expected to be minimal. A sensitivity analysis will be conducted on the primary outcome and multiple imputation by the fully conditional specification (chained equations) method will be used to impute missing outcome data.

## Ethics & Regulatory Approvals

### Declaration of Helsinki and guidelines for good clinical practice

The trial is being conducted in compliance with the principles of the Declaration of Helsinki (1996), the ICH guidelines of Good Clinical Practice (GCP) and in accordance with all applicable regulatory requirements.

### Approvals

The trial has started after gaining approval from the registered HREC of the Women’s and Children’s Health Network and other relevant regulatory authorities, including the Therapeutic Goods Administration (TGA) (Australia), the Medicines and Healthcare products Regulatory Authority (MHRA) (UK) and the European Medicines Agency (EMA). Additionally, approval of local health service Research Offices is being sought for individual trial sites.

### Participant confidentiality, data handling and record keeping

Overall responsibility for ensuring that each participant’s information is kept confidential lies with the Site Investigator. All paper documents are being stored securely and kept in strict confidence in compliance with the Data Protection Act (1998) and all trial data are being stored in line with the UK Medicines for Human Use (Clinical Trials) Amended Regulations 2006) as defined in the KHP-CTO Archiving Standard Operating Procedure (SOP).

Data entered onto the eCRFs are being downloaded for storage and analysis into an electronic database held by The University of Adelaide in which the participant is identified only by a trial specific number and their initials. The participant’s name and any other identifying details are being stored in a separate database maintained by the site which will be linked only to the database containing study data by the participant’s trial number.

Electronic files are being stored on a file server that has restricted access. Data are being processed on a workstation by authorised staff. The computer workstations access the network via a login name and password (changed regularly). No data are stored on individual workstations. Backing up is done automatically overnight to an offsite storage area.

After the trial has been completed and the reports published, the data will be archived in a secure physical or electronic location with controlled access by permission of the chief investigator and/or the TMC.

The trial data will be made available to appropriate academic parties on request from the chief investigator, in accordance with the data sharing policies of The University of Adelaide, with input from the TMC where applicable, subject to submission of a suitable study protocol and analysis plan, on publication of all initial trial results.

### Retention of personal data

Personal data will be needed to disseminate the results of the trial to the participants. Due to the nature of pregnancy research, data will be kept for a period of up to 30 years (per local site requirements) in order to follow-up on health-related issues which may become relevant in the future. At all times the personal data are to be held securely and will not be used for any other purpose.

### Protocol amendments

Any important protocol amendments (eg, changes to eligibility criteria, outcomes, analyses) will be disseminated to relevant parties (eg, TMC, DSMC, HRECs, trial participants, trial registries, regulators) as necessary.

## Assessment and reporting of safety

Assessment and reporting of adverse events and safety outcomes will follow the reporting guidelines and definitions of the NHMRC.

### Expected events

The following are considered as expected occurrences in this population of pregnant women or a result of the routine care/treatment of a participant. As such, they will be recorded on the eCRF but not reported as AEs or SAEs:

- Worsening pruritus
- Hospitalisation for worsening ICP
- Gestational diabetes/diabetes stabilisation
- Pre-eclampsia
- Admission in active labour
- Admission for cervical ripening or induction of labour
- Admission for caesarean section
- Short term (< 24 h) admission for assessment for suspected fetal compromise, including reduced fetal movements, or accelerated fetal growth and/or polyhydramnios
- Short term (< 24 h) admission for monitoring for hypertension, suspected preterm labour, or pre-labour rupture of the membranes

Such admissions are not AEs, but the underlying reason may be.

However, admission for other fetal or maternal issues, including IUGR, reduced movements, severe hypertension, seizures (eclampsia), antepartum haemorrhage, pre-term labour, pre-term premature rupture of the membranes (PPROM), psychiatric disorder, social issues, unstable lie/abnormal lie, amnioreduction, and other issues are to be reported as AEs, and may well be considered SAEs, especially if the admission is for > 24 h.

The following perinatal outcomes are pre-specified outcomes and will not be reported as AEs/SAEs:

- NNU admission
- Preterm delivery (< 37 completed weeks gestation)
- Meconium staining of the amniotic fluid or placenta
- Need for phototherapy
- Need for respiratory support – headbox oxygen

The following perinatal complications are to be reported as AEs and may be considered as SAEs:

- Neonatal seizures
- Encephalopathy treated with hypothermia
- Need for respiratory support – ventilation via an endotracheal tube or nasal CPAP
- Sepsis requiring > 5 days antibiotics with symptoms or confirmed blood or cerebro-spinal fluid (CSF) culture

The period for safety reporting is from first dose of study treatment until 6 weeks post-delivery.

### Procedures for recording AEs

Assessment for AEs are to be conducted at each study visit. All AEs occurring during the trial that are observed by the PI/delegate or reported by the participant will be recorded in the eCRF, whether or not attributed to the study drug. In recording the AE on the eCRF, whether the event was an SAE or not will be entered. All AEs determined to be an SAE must be reported following the guidelines and time frames below.

All AEs and SAEs will be followed-up to resolution with a start date and an end date recorded.

It will be left to the PI’s clinical judgement to decide whether or not an AE is of sufficient severity to require the participant’s removal from the trial. A participant may also voluntarily withdraw from treatment due to what she perceives an intolerable AE. If either of these occurs, the participant must be given appropriate care until symptoms cease or the condition becomes stable.

Unexpected serious adverse maternal and neonatal events (SAEs) will be reported to the sponsor within 24 h. The sponsor will forward these reports and their review of the SAE to the DSMC and the HREC that provided REC approval within 72 h of receipt.

### Treatment stopping rules

After inclusion of 50 women in the cohort, the DSMC will assess safety issues to ensure there is no convincing evidence of harm in either arm of the study. The DSMC will, if appropriate, make recommendations regarding continuance of the study or modification of the study protocol.

The trial management committee (TMC) has ultimate responsibility for deciding whether a trial should be stopped on safety grounds and will meet regularly to ensure recruitment milestones are met (see milestones).

If the trial is prematurely discontinued, active participants will be informed and no further participant data will be collected. The Competent Authority (CA) and all relevant HRECs will be informed within 15 days of the early termination of the trial.

### Quality control and assurance

Initiation of each participating centre will be performed by the chief investigator or their delegate once all appropriate approvals are in place. During the trial, ongoing on-site and central monitoring will be conducted. The site principal investigator (PI), research assistant and their delegates from each centre will be fully trained in protocol adherence and able to deal with site-specific issues. They will then be responsible for delivering this training to all relevant site staff prior to opening their centre for recruitment. The PI and research midwife will also promote the trial and ensure that all appropriate site staff are kept fully appraised of issues such as recruitment status, informed consent, data collection, follow-up and changing regulations, so that the necessary recruitment targets are reached within the timescale.

The trial coordinator will monitor recruitment against targets, and monitor data collection completeness and quality on a day-to-day basis.

Throughout the trial, there will be central monitoring, overseen by the TMC and DSMC, ensuring good communication between the coordinating centre and the site staff. Trial monitoring will be conducted by performing a random selection of 11 (ie 10%) participants, with at least one from each site, with an independent remote review of their eligibility criteria /consents/CRF primary outcome data.

## Trial governance

### Co-ordinating Centre

The trial co-ordinating centre is the Robinson Research Institute, The University of Adelaide, where the Chief Investigator and Trial Co-ordinator are based.

### Trial Management Committee (TMC)

The trial will be supervised on a day-to-day basis by the trial management committee, which is responsible to the trial sponsor. At each participating centre, a local PI will report to the TMC.

The TMC will meet at least monthly and consist of the CI and Trial Co-ordinator, an obstetrician and an obstetric physician from the Core Investigator group, as well as:

- Trial Statistician
- Data Manager

### Core Investigator Group (CIG)

The CIG will meet via teleconference at least four times a year. This will comprise all co-applicants and the members of the TMC.

### Data Safety Monitoring Committee (DSMC)

A data safety monitoring committee (DSMC) has been established with an independent obstetrician, a neonatologist and a statistician to ensure the wellbeing of study participants. The DSMC will review the progress and safety aspects of the trial at least annually and provide advice on the conduct of the trial to the TMC and (via the TMC) to the sponsor. The Chair of the DSMC will review SAE reports and a list of protocol breaches as received and will call a meeting of the DSMC to consider if there is evidence of potential harm from either of the interventions or from the conduct of the trial. The DSMC will otherwise review listing of all AEs, SAEs, deviations and breaches three monthly. No interim analysis will be performed, given the small sample size. The content and timings of the DSMC reviews are detailed in a DSMC Charter, which has been agreed at its first meeting.

## Publication policy/acknowledgement of contributions

The results of the trial will be presented at an international meeting, likely the biennial meeting of the International Society of Obstetric Medicine, and will be offered for publication to leading international clinical journals. They will be disseminated to important stakeholders, including the consumer group, ICP Support, and transmitted to those responsible for creation and updating of clinical guidelines for patient management, such as the Royal College of Obstetricians and Gynaecologists, and the Royal Australian and New Zealand College of Obstetricians and Gynaecologists.

The success of the trial depends on a large number of midwives, obstetricians and participants. Credit for the trial findings will be given to all those who have collaborated and participated in the study including all local coordinators and collaborators, members of trial committees and study staff.

## Discussion

Our study will be the first to examine the outcomes of treatment specifically in the severe early onset form of ICP, comparing “standard” UDCA therapy with rifampicin, and so be able to provide for the first-time high-quality evidence for use of rifampicin in severe ICP. It will also allow an assessment of feasibility of a future trial to test whether elective early delivery in severe ICP is beneficial.

Recruitment is underway, despite the difficulties of the current pandemic, which have delayed many centres from embarking on new research that is unrelated to COVID-19 or even considering undertaking it. We applaud all the research governance offices who have been willing to enable this important trial, which has the potential to impact on the lives of the women affected by a rare disorder of pregnancy and, in due course, to enable further trials to examine the bigger and more difficult questions of how to improve pregnancy outcome.

## Supplementary Information

**Additional file 1:.** The approved master PICF for the TURRIFIC trial is attached as an appendix, together with a copy of the DSMC charter and the SPIRIT checklist.

## Data Availability

The dataset will be available to appropriate academic parties on request from the Chief Investigator, William Hague, in accordance with the data sharing policies of The University of Adelaide, with input from the coinvestigator group where applicable, subject to submission of a suitable study protocol and analysis plan, on publication of all initial trial results.

## Abbreviations

AE: Adverse Event
ALT: Alanine transaminase
ANA: Anti-nuclear antibody
ANZCTR: Australian New Zealand Clinical Trials Register
APTT: Activated Partial Thromboplastin Time
BA: Bile acids
BRIC: Benign Recurrent Intrahepatic Cholestasis
BSEP: Bile Salt Export Pump
CA: Competent Authority
CI: Confidence Interval
CIG: Core Investigator Group
CMV: Cytomegalovirus
CONSORT: CONsolidated Standards Of Reporting Trials
CPAP: Continuous Positive Airway Pressure
CSF: Cerebro-spinal fluid
CTG: Cardiotocography
DSMC: Data Safety Monitoring Committee
EBV: Epstein-Barr virus
EudraCT: European Union Drug Regulating AuthoritiesClinical Trials database
eCRF: electronic Case Record Form
EEG: Electroencephalogram
EMA: European Medicines Agency
FXR: Farnesoid X receptor
GCP: Good Clinical Practice
GDM: Gestational Diabetes Mellitus
GEE: Generalised Estimating Equation
GGT: Gamma Glutamyl Transferase
GRADE: Grading of Recommendations, Assessment, Development and Evaluations
GROW: Gestation Related Optimal Weight
HIV: Human Immunodeficiency Virus
HREC: Human and Research Ethics Committee
HSV: Herpes simplex virus
ICH: International Council for Harmonisation of Technical Requirements for Pharmaceuticals for Human Use
ICP: Intrahepatic Cholestasis of Pregnancy
ICU: Intensive Care Unit
INR: International Normalised Ratio
IRAS: Integrated Research Approval System
ISSHP: International Society for the Study of Hypertension in Pregnancy
KHP-CTO: King’s Health Partners Clinical Trials Office
LFTs: Liver Function Tests
LKM: Liver Kidney Microsomal
MD: Mean difference
MHRA: Medicines and Healthcare products Regulatory Authority
MRFF: Medical Research Future Fund
NHMRC: National Health and Medical Research Council
NNU: Neonatal unit
OGTT: Oral glucose tolerance test
PI: Principal Investigator
PICF: Patient Information and Consent Form
PITCH: Pregnancy Intervention Trial in Cholestasis
PITCHES: Phase 3 trial in IntrahepaTic CHolestasis of pregnancy to Evaluate urSodeoxycholic acid
PPH: Postpartum haemorrhage
PPROM: Pre-term Premature Rupture Of the Membranes
RCT: Randomised controlled trial
REDCap: Research Electronic Data Capture
RIF: Rifampicin
RR: Risk ratio
SAE: Serious Adverse Event
SD: Standard Deviation
SOP: Standard Operating Procedure
SPIRIT: Standard Protocol Items: Recommendations for Interventional Trials
STRIDER: Sildenafil TheRapy In Dismal Prognosis Early-onset Fetal Growth Restriction
TB: Tuberculosis
TGA: Therapeutic Goods Administration
TMC: Trial Management Committee
UDCA: Ursodeoxycholic acid
UK: United Kingdom
VAS: Visual analogue scale
WHO: World Health Organisation
