# Supplementary material for: A multi-centre, open label, randomised, parallel-group, superiority Trial to compare the efficacy of URsodeoxycholic acid with RIFampicin in the management of women with severe early onset Intrahepatic Cholestasis of pregnancy: the TURRIFIC randomised trial
Source: BMC Pregnancy Childbirth. 2021 Jan 12;21:51. doi: 10.1186/s12884-020-03481-y (PMC7802989; doi:10.1186/s12884-020-03481-y)
Supplement: Supplementary file 1 — Additional file 1:. The approved master PICF for the TURRIFIC trial is attached as an appendix, together with a copy of the DSMC charter and the SPIRIT checklist. [file 12884_2020_3481_MOESM1_ESM.zip › TURRIFIC DSMC Charter V2 Final 210920R2.pdf]

A randomised Trial of URsodeoxycholic acid versus RIFampicin in severe early onset Intrahepatic Cholestasis of pregnancy: the TURRIFIC study.

Trial Identifiers
ACTRN: 12618000332224p
HREC No: HREC/18/WCHN/36
EudraCT number: 2018-004011-44
IRAS 272398

Sponsor
The University of Adelaide,
ADELAIDE SA 5005
AUSTRALIA

Funder
National Health and Medical Research Council (NHMRC) Medical Research Future Fund (MRFF) Lifting Clinical Trials and Registries Capacity (LCTRC) Grant Opportunity.

Chief Investigator
Professor Bill Hague
Women's and Babies' Division
Women's and Children's Health Network
72 King William Rd,
NORTH ADELAIDE SA 5006
Tel: +61 411 114 575
bill.hague@adelaide.edu.au

Trial Co-ordinating Centre
Robinson Research Institute
Discipline of Obstetrics and Gynaecology
The University of Adelaide
55 King William Rd,
NORTH ADELAIDE SA 5006
Tel: +61 8 8313 1338

Data Safety Monitoring Committee Charter

(adapted from the DAMOCLES model, Lancet 2005)

Prepared and authorised by:
Professor Bill Hague: Chief Investigator

Signature: Date:

CONTENT	CHARTER DETAILS
Introduction	
**Name of trial**	A Randomised Trial of URsodeoxycholic acid versus RIFampicin in women with severe early onset Intrahepatic Cholestasis of pregnancy (the TURRIFIC study)
**TRIAL INTERVENTION**	Rifampicin
**TRIAL COMPARATOR**	Ursodeoxycholic acid
Objectives of trial, including interventions being investigated	PRIMARY OBJECTIVE<br>1. To evaluate whether the administration of rifampicin (RIF) improves pruritus more in women with severe early onset intrahepatic cholestasis of pregnancy (ICP) than ursodeoxycholic acid (UDCA)<br><br>Exploratory objectives:<br>2. To evaluate whether RIF, compared with UDCA, improves maternal liver chemistry in severe early onset ICP, including serum bile acids (BA) and serum transaminase concentrations<br>3. To evaluate whether RIF, compared with UDCA, improves serum and urine pruritogen concentrations in severe early onset ICP, including serum autotaxin and progesterone sulphated metabolites (PSM), and urine glucuronidated 6α-hydroxylated BA<br>4. To evaluate influence of treatment with (i) UDCA, (ii) RIF, (iii) both drugs on the gut microbiota and gut metabolites (fecal profiles of individual BA, short chain fatty acids (SCFA) and related metabolites) in women with ICP<br>5. To investigate whether the enterotype prior to treatment influences alterations in gut metabolites when given specific drugs<br>6. To ascertain the relationship between altered fecal metabolites and improvement in severity of cholestasis quantified by degree of hypercholanaemia, hepatic impairment or severity of pruritus<br>7. To use UPLC-MS/MS to compare serum and urine profiles of PSM in women with ICP, depending on the treatment modality<br>8. To investigate whether the maternal genome influences the responses to treatment of women with ICP<br>9. To investigate whether viral exposure during pregnancy influences the responses to treatment of women with ICP
Outline of scope of charter	The purpose of this document is to describe the membership, terms of reference, roles, responsibilities, authority and decision-making of the DSMC for the TURRIFIC study. This includes the timing of meetings, methods of providing information to and from the DSMC, frequency and format of meetings, statistical issues and relationships with other committees.

Roles and responsibilities

The aims of the committee	To protect and serve TURRIFIC study patients regarding safety and to assist and advise the Chief Investigator (CI) and Trial Management Committee (TMC), so as to protect the validity and credibility of the trial.<br><br>To safeguard the interests of TURRIFIC study patients, assess the safety and efficacy of the interventions during the trial, and monitor the overall conduct of the TURRIFIC study.
Terms of reference	The DSMC will receive and review the progress and accruing data of the TURRIFIC study and provide advice on the conduct of the trial to the TMC. The review of the trial's progress will include data quality, and main endpoints including safety data.<br><br>The DSMC shall inform the Chair of the TMC if, in their view:<br><br>(i) the results are likely to convince a broad range of clinicians, including those supporting the trial and the general clinical community, that one trial arm, or a subset of the trial population, is clearly indicated or contraindicated, and that there is a reasonable expectation that this new evidence would materially influence patient management;<br><br>or<br><br>(ii) it becomes evident that no clear outcome will be obtained.
Specific roles	assessment of data quality, including completeness and accuracy (and by so doing encourage collection of high-quality data)<br><br>monitoring of participant and investigator compliance with the protocol<br><br>monitoring of evidence for treatment differences in the main efficacy endpoints<br><br>monitoring of evidence for treatment harm (eg toxicity data, SAEs, deaths)<br><br>review of all reports of suspected unexpected serious adverse reactions (SUSARs) provided by the trial team<br><br>deciding whether to recommend that the trial continues to recruit participants or whether recruitment should be terminated either for everyone or for some treatment groups and/or some participant subgroups<br><br>suggestion of additional data analyses<br><br>provision of advice on protocol modifications suggested by the TMC (eg inclusion criteria, trial endpoints, or sample size)<br><br>monitoring of continuing appropriateness of patient information<br><br>monitoring of compliance with previous DSMC recommendations<br><br>consideration of the ethical implications of any recommendations made by the DSMC

assessment of the impact and relevance of external evidence

maintenance of confidentiality of all trial information that is not in the public domain

protection of the validity and scientific credibility of the trial

Early in the trial

DSMC input into the protocol

All potential DSMC members will have been given sight of the protocol/outline before agreeing to join the committee.

If a potential DSMC member were to have had major reservations about the trial (eg, the protocol or the logistics), they should have reported these to the CI and may have decided not to accept the invitation to join. DSMC members shall be independent and constructively critical of the ongoing trial, but also supportive of the aims and methods of the trial.

Meetings

It was recommended that, if possible, the DSMC would meet early in the course of the trial, to discuss the protocol, the trial, any analysis plan, future meetings, and to have the opportunity to clarify any aspects with the CI and the trial coordinator.

The DSMC should meet within one year of recruitment commencing.

Consideration would be given to an initial "dummy" report, including the use of shell (empty) tables, to familiarise the DSMC members with the format that will be used in the reports.

Any specific regulatory issues

The DSMC are to be aware of any regulatory implications of their recommendations.

Need for contracts

DSMC members will not formally sign a contract but will formally register their assent to join the group by confirming (1) that they agree to be on the DSMC and (2) that they agree with the contents of this Charter. Any competing interests will be declared at the same time. Members will complete and return the form in Annex 1. All members and observers attending any part of subsequent meetings are to sign a confidentiality agreement on the first occasion they attend all or part of a meeting (Annex 2).

Composition of the DSMC

Members

The DSMC includes an obstetrician and a neonatologist, both experienced in the management of women with ICP and their offspring, together with an independent statistician, experienced in the assessment and analysis of clinical trials.

The members will not be involved with the trial in any other way or have some competing interest that could impact on the trial. Any competing interests, both real and potential, are to be declared.

Although members may well be able to act objectively despite such connections, complete disclosure enhances credibility. The

short competing interest form is to be completed and returned by the DSMC members to the trial coordinator (Annex 1).

The members of the DSMC for this trial are:

- Dr Wessel Ganzevoort (Obstetrician, AMC Amsterdam)
- A/Prof Chad Andersen (Neonatologist, WCHN Adelaide)
- Dr Emma Knight (Biostatistician, South Australian Health and Medical Research Institute)

The Chair of the DSMC will be chosen by the DSMC members amongst themselves. The Chair is expected to facilitate and summarise discussions.

Responsibilities of the trial statistician	The trial statistician has responsibility for producing reports to the DSMC but will not participate in DSMC meetings, unless requested. The DSMC statistician will guide the DSMC through the reports provided by the trial statistician.
Responsibilities of the trial coordinator	The trial coordinator may help the trial statistician to produce the non-confidential sections of the DSMC report. The trial coordinator may attend open sessions of the meeting.
Responsibilities of the CI	The CI may be asked, and will be available, to attend open sessions of the DSMC meeting. The other TMC members will not usually be expected to attend but may attend open sessions when necessary (See Section 6. Organisation of DSMC Meetings).
<u>Relationships</u>	A diagram is included in this charter (Figure 2) to illustrate the relationships between the trial committees and the sponsor.<br><br>The TMC is responsible for the overall supervision of the trial progress and will make executive decisions about the trial during these meetings.<br><br>The DSMC meets to review all data collected. In addition, a DSMC meeting may be triggered for safety reasons and under 'specific roles of the DSMC' (as above).<br><br>If a DSMC meeting is convened for reasons other than those described above, then their role will be in an advisory capacity to the TMC.
<u>Payments to DSMC members</u>	No payments or rewards will be given to DSMC members.<br><br>DSMC members are not to use interim results to inform trading in pharmaceutical shares, and careful consideration is to be given to trading in stock of companies with competing products.
<u>Organisation of DSMC meetings</u>	The exact frequency of meetings will depend upon any statistical plans specified and otherwise on trial events.<br><br>Notwithstanding this, the DSMC will receive an update from the TMC regarding study conduct at least every 3 months.

	The wishes of the DSMC and needs of the trial coordinating team are to be considered when planning each meeting. The DSMC will meet at least yearly.<br><br>An unplanned DSMC meeting may be called by the Chair or requested by the TMC if there is an emergency concern on the safety of participants.
Style of meetings	Although face to face meetings are preferable, video teleconferencing (ZOOM or equivalent) will be used for the DSMC meetings, given the exigencies of intercontinental travel.
Open/closed sessions	DSMC meetings may contain a mixture of open and closed sessions.<br><br>Closed sessions:<br>Only DSMC members, and others whom they specifically invite, e.g. the trial statistician, are to be present in closed sessions.<br><br>Open sessions:<br>All those attending closed sessions may be joined by the CI, other principal investigators, the trial coordinator, and sometimes also representatives of the sponsor, funder, or regulator, as relevant.
<u>Suggested DSMC meeting format</u>	Open session: Introduction and any "open" parts of the report<br><br>Closed session: DSMC discussion of "closed" parts of the report<br><br>Open session (if required): Discussion with other attendees on any matters arising from the previous session(s).<br><br>Closed session: any extra closed session as required.
<u>Confidentiality and communication procedures for trial documents</u>	<u>Open sessions</u>:<br>Accumulating data relating to recruitment and data quality (e.g., data return rates, sample collection) will be presented. Any toxicity details, based on pooled data, will be presented and total numbers of events for the primary outcome measure and other outcome measures may be presented, at the discretion of the DSMC.<br><br><u>Closed sessions:</u><br>In addition to all the material available in the open session, the closed session material will include efficacy and safety data by treatment group.
<u>Blinding and Confidentiality</u>	Members of the DSMC will not be blinded as to treatment allocation.<br><br>The confidential accumulating data and interim analysis by treatment allocation will only be seen by the DSMC members.<br><br>DSMC members do not have the right to share confidential information with anyone outside the DSMC, including the CI.<br><br>Identification and circulation of external evidence (e.g., from other trials/ systematic reviews) is not the responsibility of the DSMC members. The CI, TMC and the trial coordinator will

	collate any such information for the presentation in an open session.
Communication of DSMC decisions/recommendations	The DSMC will report its decisions/recommendations in writing to the CI and the TMC chair. This will be copied to the trial statistician (and/or trial coordinator) and, if possible, will be sent via the trial statistician (or trial coordinator) in time for consideration at a TMC meeting where necessary. If the trial is to continue largely unchanged, then it may be useful for the report from the DSMC to include a summary paragraph suitable for trial promotion purposes. (See Annex 3.)<br><br>In its communications, the DSMC will be careful not to relay any unnecessary information to the TMC.
Provision of data to the DSMC	For planned DSMC meetings, it will usually be helpful for the DSMC to receive the progress report at least 2 weeks before any meetings. For unplanned meetings, it may be preferable for all papers to be brought to face-to-face/Zoom meetings by the trial statistician; time will then be needed for DSMC members to assimilate the data/report.<br><br>DSMC members are to store the papers safely after each meeting so that they may check the next report against them. After the trial is finally reported, DSMC members are to destroy all interim reports. A copy of all the reports will be held in the Trial Master File.

<u>Decision-making</u>

Possible options open to the DSMC	Decisions/recommendations may include:<br><br>No action needed; trial to continue as planned<br><br>Early stopping due, for example, to clear benefit or harm of a treatment, futility, or external evidence<br><br>Stopping recruitment within a subgroup<br><br>Extending recruitment or extending follow-up<br><br>Sanctioning and/or proposing protocol changes
Interim analyses	No interim analyses are scheduled to occur during the trial.<br><br>However, the TMC will provide the DSMC with study data (unblinded in closed meeting) after inclusion of 50 women as set out in the Protocol, Version 9.
How decisions or recommendations will be reached within the DSMC	The DSMC chair shall encourage consensus. It is important that the implications (e.g. ethical, statistical, practical, financial) for the trial be considered before any recommendation is made.<br><br>Every effort will be made for the DSMC to reach a unanimous decision. If the DSMC cannot achieve this, a vote may be taken, although details of the vote will not be routinely included in the report to the TMC, as these may inappropriately convey information about the state of the trial data.

<u>Reporting</u>

Reports from the DSMC	These are to be communicated by letter to the CI and the TMC chair, delivered within 3 weeks for planned meetings, and as promptly as possible following unplanned/triggered meetings.<br><br>A copy of the DSMC recommendations/decision letters will be stored in the trial master file.
Minutes and reports of meetings	Minutes of any open meetings will be recorded electronically and transcribed by the trial coordinator. Minutes will be finalised upon signature of the chairperson and maintained by the sponsor in accordance with applicable statutory regulations.<br><br>The minutes of closed sessions will be recorded electronically and transcribed by an independent DSMC designee. Minutes from closed sessions will be recorded separately from the minutes of open sessions and stored securely by the sponsor. Closed session minutes, finalised by signature of the DSMC chair, will be maintained in confidence and retained until discarded in accordance with applicable statutory regulations.<br><br>Following each meeting, a report separate from the minutes of the open and closed sessions is to be sent to the sponsor/TMC describing the DSMC recommendations and rationale for such.
Disagreements	If the DSMC has serious problems or concerns with the TMC decisions or *vice versa*, a meeting of these groups should be held. The information to be shown will depend upon the action proposed and the DSMC's concerns. Depending on the reason for the disagreement, confidential data may have to be revealed to all those attending such a meeting. The meeting is to be chaired by a senior member of the local Clinical Trials Unit or an external expert who is not directly involved with the trial.

<u>After the trial</u>

Publication of results	At the end of the trial there may be a meeting to allow the DSMC to discuss the final data with the key members of the TMC and to give advice about data interpretation.<br><br>The DSMC may wish to see a statement that the trial results will be published in a correct and timely manner.
Information about the DSMC members to be included in published trial reports	DSMC members will be named, and their affiliations listed, in the main report, unless they explicitly request otherwise. A brief summary of the timings and conclusions of DSMC meetings will be included in the body of this paper.
DSMC comment on, and approval of, proposed publications, especially in relation to any recommend-ations for ending the trial.	The DSMC will be given the opportunity to read and comment on publications before submission.
Constraints on disclosure of DSMC discussions after publication of trial results	The DSMC may discuss issues from their involvement in the trial 12 months after the primary trial results have been published, or sooner with permission from the TMC.

FIGURES AND APPENDICES

Figure 1. Trial Flow chart

Figure 2. Relationship of trial committees, including DSMC and TMC

Annex 1: Agreement and potential competing interests form

Annex 2: Agreement and confidentiality agreement for observers

Annex 3: Suggested report proforma from DSMC to TMC

Annex 4: Trial Contacts

Annex 5: Summary of changes from previous versions

Figure 1. Trial Flow chart: Planned Enrolment of subjects

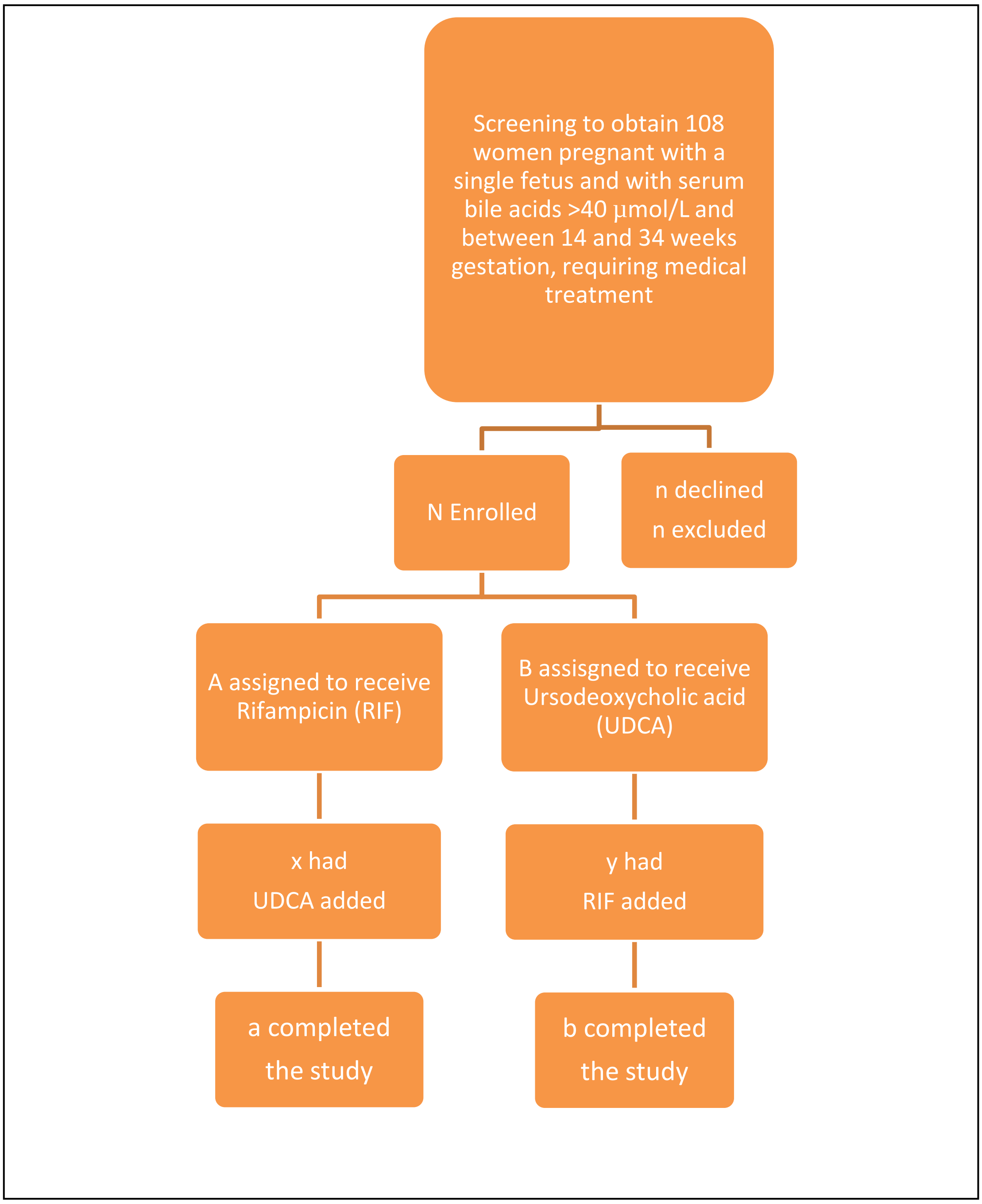

Figure 2. Relationship of trial committees, including DSMC and TMC

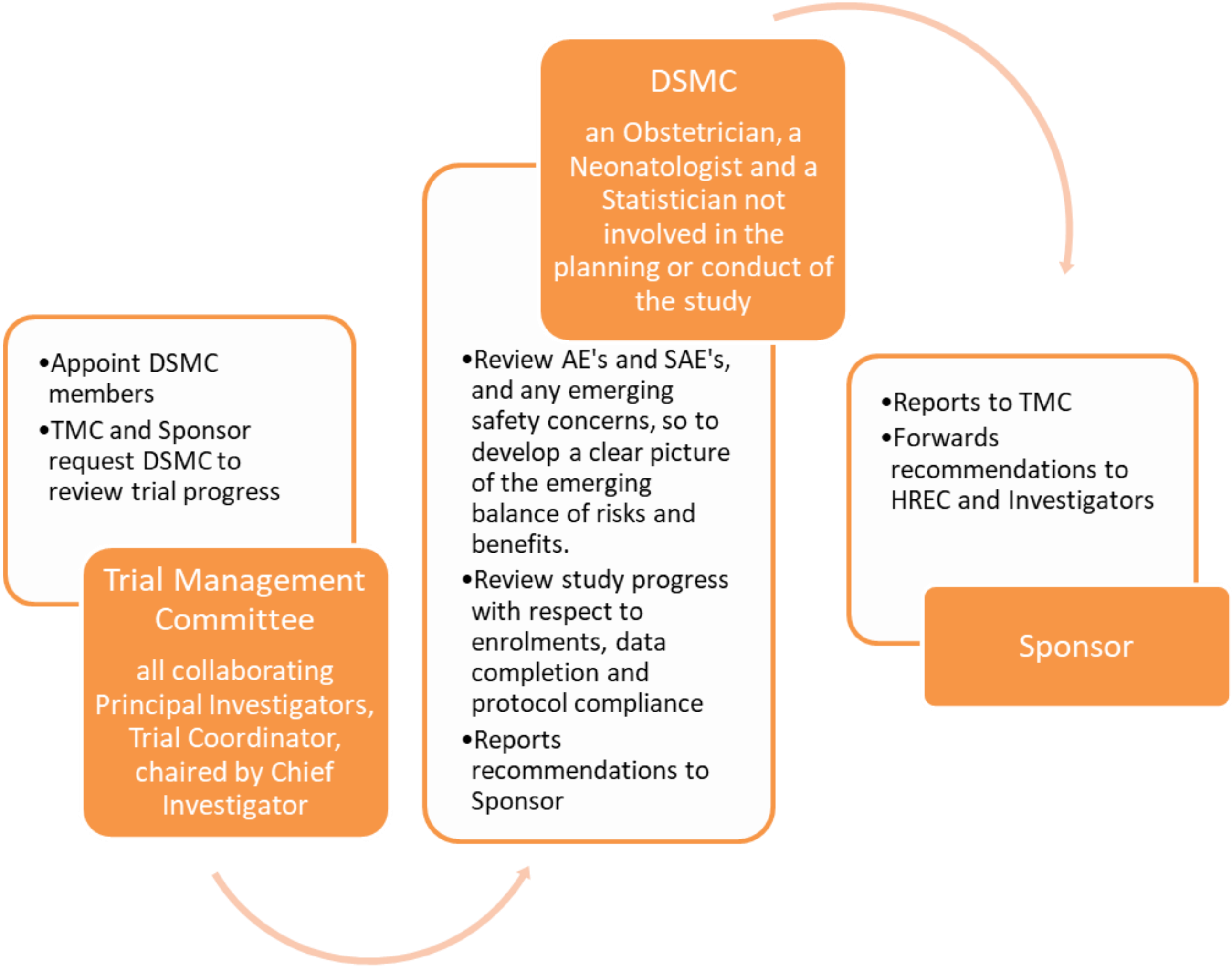

Annex 1: Agreement and potential competing interests form

A randomised Trial of URsodeoxycholic acid versus RIFampicin in severe early onset Intrahepatic Cholestasis of pregnancy: the TURRIFIC study

SPONSOR: The University of Adelaide

Data Safety Monitoring Committee agreement and conflict of interest form

I, (insert name) ________________________________ agree to act as a member of the Data Safety Monitoring Committee for this study.

I agree to contribute to the DSMC deliberations and to report on study safety and progress to the Sponsor, as outlined in this Charter.

I agree to keep confidential information that I have access to in the performance of my role, especially in relation to interim study outcomes.

I disclose the following conflicts of interest:

- ☐ Nil
- ☐ Stock ownership in any commercial company involved
- ☐ Stock transaction in any commercial company involved (if previously holding stock)
- ☐ Consulting arrangements with the Sponsor
- ☐ Frequent speaking engagements on behalf of the intervention
- ☐ Career tied up in a product assessed by the trial
- ☐ Hands-on participation in the trial
- ☐ Involvement in the running of the trial
- ☐ Emotional involvement in the trial
- ☐ Intellectual conflict, e.g. strong prior belief in the trial's experimental arm
- ☐ Involvement in regulatory issues relevant to the trial procedures
- ☐ Investment (financial or intellectual) in competing products
- ☐ Involvement in the publication

Please provide details of the competing interests:_____________________________________

____

____

The avoidance of any perception that members of the DSMC may be biased in any way is important for the credibility of the decisions made by the DSMC and for the integrity of the trial. It is therefore important that any competing interest be disclosed. This simple disclosure may be sufficient. Where it is deemed that the conflict is too great, the DSMC member may need to either remove the conflict or cease participation in the DSMC.

Name:____

Signed:_______________________________________ Date:______________________

Annex 2: Agreement and confidentiality agreement for observers

A randomised Trial of URsodeoxycholic acid versus RIFampicin in severe early onset Intrahepatic Cholestasis of pregnancy: the TURRIFIC study

SPONSOR: The University of Adelaide

Confidentiality agreement

I, (insert name) ____________________________________ agree to keep confidential information that I have access to in the attendance at any TURRIFIC trial Data Safety Monitoring Committee meeting especially in relation to interim study outcomes.

Name:____

Signed:____ Date:________________________

Annex 3: Suggested report proforma from DSMC to TMC

[Insert date]

To: Chair of TURRIFIC study Trial Management Committee

Dear [Chair of TMC],

The Data Safety Monitoring Committee (DSMC) for the TURRIFIC study met on [insert date] to review progress and interim accumulating data. [List Members] attended the meeting and reviewed the report.

The trial question remains important and, based on the data reviewed at this stage, we recommend continuation of the trial according to the protocol [specify protocol version and date] with no changes.

Or (for example) The issue of [enter details] is a safety concern. We recommend [detail suggested protocol amendment] to the protocol [specify version and date].

We shall next review study progress and data on [provide approximate meeting date]

Yours sincerely,

[Name of DSMC Chair]

Chair, TURRIFIC Study DSMC

On behalf of the DSMC (members listed below)
Wessel Ganzevoort (Obstetrician)
Chad Anderson (Neonatologist)
Emma Knight (Statistician)

Annex 4: Trial Contacts

Chief Investigator: Prof Bill Hague	+61 411 114 575
	bill.hague@adelaide.edu.au
Trial Coordinator: Suzette Coat	+61 883 131 338
	suzette.coat@adelaide.edu.au
Trial Statistician: Dr Jennie Louise	jennie.louise@adelaide.edu.au

Annex 5: Summary of changes from previous versions

This table to be amended each time the DSMC Charter is amended.

1. Add new row at top of table to insert Current Version and date. All previous Version numbers and dates to remain in table (no deletions should be made).
2. Amendments for revised Version to be added in table below. New rows may be added to accommodate details of the changes. No rows should be deleted. Briefly detail the amendment, referencing page number and heading the amendment comes under and reasoning for the change.

Current Version #	2	Version Date	21 September 2020	Approved by:	
Previous Version #	1	Version Date	24 April 2020	Approved by:	
Version 2 amendments:	Insertion of second to last paragraph on page 5, “Notwithstanding…..”<br>Last paragraph, page 5, *et passim*, “should” replaced with “will” or “are to”, as appropriate.<br>Insertion of paragraph to page 7, “Interim analysis” section, “However, the TMC will provide the DSMC….”<br>Main body of document reformatted as a table to facilitate future amendments.				
