# Supplementary material for: A multi-centre, open label, randomised, parallel-group, superiority Trial to compare the efficacy of URsodeoxycholic acid with RIFampicin in the management of women with severe early onset Intrahepatic Cholestasis of pregnancy: the TURRIFIC randomised trial
Source: BMC Pregnancy Childbirth. 2021 Jan 12;21:51. doi: 10.1186/s12884-020-03481-y (PMC7802989; doi:10.1186/s12884-020-03481-y)
Supplement: Supplementary file 1 — Additional file 1:. The approved master PICF for the TURRIFIC trial is attached as an appendix, together with a copy of the DSMC charter and the SPIRIT checklist. [file 12884_2020_3481_MOESM1_ESM.zip › TURRIFIC PICF V5 Protocol paperR2.docx]

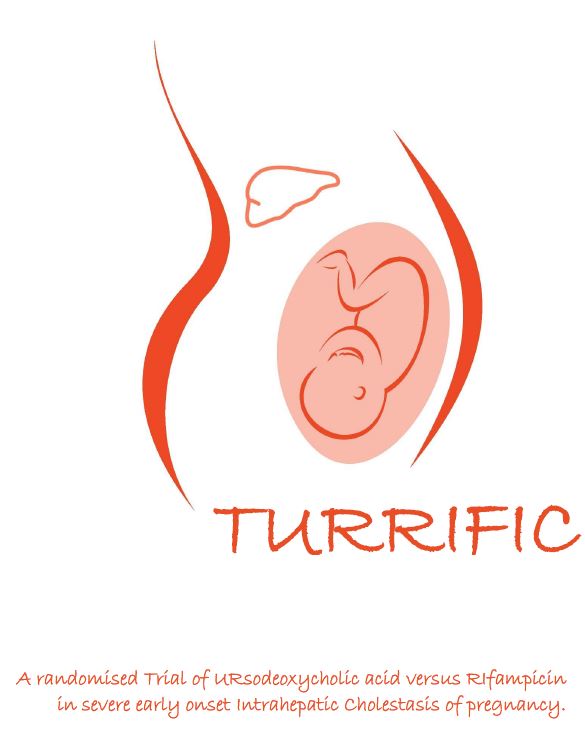


*Insert Header with institution’s name or institution’s letterhead*

**Participant Information Sheet and Consent Form**

| **Title** | *A randomised Trial of URsodeoxycholic acid versus RIFampicin in severe early onset Intrahepatic Cholestasis of pregnancy: the TURRIFIC study* |
| --- | --- |
| **Short Title** | *TURRIFIC* |
| **Lay Title** | *A Trial comparing URsodeoxycholic acid with RIFampicin in severe early onset Intrahepatic Cholestasis of pregnancy: the TURRIFIC study* |
| **Project Sponsor** | *University of Adelaide* |
| **Principal Investigator** | *[enter PI name]* |
| **Associate Investigator(s)** | *[enter AI names]* |
| **Location** | *[enter location name]* |

**The “TURRIFIC trial”**

**Part 1 What does my participation involve?**

1. **Introduction**

You are invited to take part in this research project. This is because you have severe early onset Intrahepatic Cholestasis of Pregnancy (ICP). The research project is testing a new treatment for ICP. The new treatment is called rifampicin, a drug commonly used for treatment of infections, such as tuberculosis (TB), including in pregnant women.

This Participant Information Sheet/Consent Form tells you about the research project. It explains the tests and treatments involved. Knowing what is involved will help you decide if you want to take part in the research.

Please read this information carefully. Ask questions about anything that you don’t understand or want to know more about. Before deciding whether or not to take part, you might want to talk about it with a relative, friend or your local doctor.

Participation in this research is voluntary. If you don’t wish to take part, you don’t have to. You will receive the best possible care whether or not you take part.

If you decide you want to take part in the research project, you will be asked to sign the consent section. By signing it, you are telling us that you:

- Understand what you have read
- Consent to take part in the research project
- Consent to have the tests and treatments that are described

Consent to the use of your personal and health information as described.

You will be given a copy of this Participant Information and Consent Form (PICF) to keep.

**2 What is the purpose of this research?**

Severe early onset (before 34 weeks of gestation) intrahepatic cholestasis of pregnancy (ICP), a recognised rare disease of pregnancy, is associated with stillbirth, preterm birth and medical problems in newborn babies. The best way to treat and manage women who are diagnosed with this condition is not known. Evidence from carefully designed clinical trials is urgently needed. The current study represents a unique opportunity to establish evidence that will help to inform doctors and healthcare professionals around the world caring for women with this condition. We have assembled an international team of clinical researchers to test for the most effective treatments.

Standard treatment at [hospital name] for women with ICP includes ursodeoxycholic acid (UDCA), which is not always 100% effective, especially in women with severe disease. UDCA has also been used outside of pregnancy for people with gallstones and for people with some other liver disorders. Rifampicin is a drug that has been used for the treatment of infections, such as tuberculosis, including in pregnant women and babies. It has also been found to be effective in treating the itch associated with cholestatic disorders outside of pregnancy, especially in people needing palliative care. There is a small case series where rifampicin has been used “off label” (i.e. without approval for use for treating a medical condition) in severe ICP, but there are no formal trials to compare it with UDCA, either in or out of pregnancy.

Medications, drugs and devices have to be approved for use by the Australian Federal Government. Rifampicin is approved in Australia to treat infections, such as tuberculosis. However, it is not approved to treat ICP. Therefore, rifampicin is an experimental first line treatment for ICP. This means that it must be tested to see if it is an effective treatment for ICP.

This study is designed to compare the effectiveness of rifampicin, as an experimental first-line treatment for severe early onset ICP, against UDCA, which is the current standard first-line treatment for ICP.

**3 What does participation in this research involve?**

You will be eligible for this study if you are more than 14 weeks pregnant and you develop ICP before 34 weeks gestation, and your ICP is or becomes severe, defined as a high bile acid concentration in the blood (40 µmol/L or greater).

You will be participating in a randomised controlled research project. Sometimes we do not know which treatment is best for treating a condition. To find out, we need to compare different treatments. We put people into groups and give each group a different treatment. The results are then compared to see if one is better. To try to make sure the groups are the same, each participant is put into a group by chance (random).

In this study, you will be offered a one in two chance of receiving the experimental first-line treatment for severe ICP, rifampicin, or the standard treatment, ursodeoxycholic acid (UDCA). If you are allocated to rifampicin, you will be prescribed a standard dose of 300mg (one tablet) twice daily, which will stay the same for the duration of the study. If you are allocated to UDCA, you will be commenced on 450-1000mg daily as per the usual protocol at [hospital] in single or divided doses: the dose of UDCA will be increased every 3-14 days if there is no improvement in your itch or in the associated blood tests to a maximum of 2000mg per day. If you are already taking UDCA for mild ICP, and then develop severe ICP, you will be asked to come off treatment for four to seven days to allow for a washout period and baseline assessment before randomisation to continuing treatment with UDCA or switching to rifampicin. During this washout period, there is the small chance of some worsening of your itch back to how it was before UDCA was started, although there is also the possibility that the itch may have already improved. It is an “open study”, meaning that you will know what medication you are taking. In addition, taking rifampicin tends to turn body fluids an orange colour.

The only restrictions to taking part in the study are:

- A decision has already been made for your delivery within the next 48 hours
- You are allergic to any component of the UDCA or rifampicin tablets
- You are taking medication that is known to have a significant interaction with rifampicin. If you are taking medication, it will be discussed with you by the research worker.
- You are carrying more than one baby
- You have active Hepatitis A or Hepatitis B infection, or are a Hepatitis B or C carrier
- You have the pregnancy disorder pre-eclampsia (high blood pressure and other associated features)
- You are known to have α-1-antitrypsin deficiency, autoimmune hepatitis, or other known liver disorder
- You are already taking medication that can cause changes in your liver tests
- You do not wish to have your baby given the standard Vitamin K injection at birth

If you agree to take part, you will be invited to attend an appointment of approximately one hour duration at the [insert name] Hospital. At this appointment we will:

- Discuss the study and any questions you have about it,
- If you are willing to participate you will be asked to sign the consent form. No study assessments will be conducted before you have signed the consent form.
- Collect “baseline data”: we will collect some basic demographic information about you, such as your age and where you live, and ask questions about your health and pregnancy history. We will also ask about the health of your family members.
- We will ask you to indicate how bad your itch has been in the last 24 hours.
- We will be recording your blood test results for serum bile acid concentration, liver function and blood clotting from within seven days before study entry.
- We will enter some information about you into our study database and will then randomly allocate your study treatment to you. Once we have your treatment allocation we will submit a prescription to the hospital pharmacy for your treatment.

**Subsequent study visits:**

7 days after study entry and then on a monthly basis.

You will be invited to return to see us one week after you join the study. Where possible we will arrange this on the same day near to the time you have a clinic visit. At this visit we will:

- Discuss the study and any questions you have about it,
- Ask you to indicate how bad your itch has been in the last 24 hours.
- Record your routine blood test results for serum bile acid concentration, liver function and blood clotting after seven days of study treatment.
- Ask you about any reaction you might have had to your study treatment, any illnesses you have had since we last saw you, and any medication you may have been prescribed in the meantime.

Thereafter, on a monthly basis until 28 weeks of your pregnancy, and then every week where possible, we will see you when you attend the hospital for clinic visits. At each of these visits we will:

- Ask you to indicate how bad your itch has been in the last 24 hours.
- Record your routine blood test results for serum bile acid concentration, liver function and blood clotting each time.
- Ask you about any reaction you might have had to your study treatment, any illnesses you have had since we last saw you, and any medication you may have been prescribed in the meantime.

Additionally, we will record your blood pressure at each visit. We will also be recording whether you develop complications, such as gestational diabetes, and how they are being treated.

At birth, with your consent we will examine the placenta, as is usually done at this hospital for women with ICP, using a standardised procedure to report on its apparent health. We will also collect a very small sample, a “slide”, to be sent to a Placental Pathologist at the Women’s and Children’s Hospital in Adelaide, to examine the micro-structure of the sample, which, if there are any problems experienced by the baby, will help to interpret them.

After your baby is born, we will collect information about your health and your baby’s health at birth from the hospital medical record. This will ensure that all appropriate factors that might impact on the outcome of a drug trial can be reviewed and taken into account.

You will be asked to return for a follow-up visit at 6 weeks after the birth of your baby to check that the ICP has cleared and to provide any initial results from the trial that are available. We will write to you with a summary of results, once the study has been completed, and offer an appointment for a face to face discussion of the outcome. This may not be for a year or more after the completion of your pregnancy.

This research project has been designed to make sure the researchers interpret the results in a fair and appropriate way, and to avoid study doctors or participants making inappropriate decisions on inadequate evidence.

There are no additional costs associated with participating in this research project, but nor will you be paid. All tests and medical care required as part of the research project will be provided to you free of charge.

The trial medications will be provided at no cost to you. You will, however, need to pay for any other or extra medications, as usual, according to hospital policy, for which you will incur a standard prescription charge, depending on your ability to pay.

You may be reimbursed for any reasonable travel, parking, meals and other expenses associated with a research project visit not associated with your standard care.

If you decide to participate in this research project, with your permission the study doctor will inform your local doctor. With your permission also, information about your condition will be provided to your local doctor at the end of the pregnancy, and advice given about management in any future pregnancy, should this occur.

**4 What do I have to do?**

If you agree to participate in the trial, we will need to have an appointment with you. In this appointment we will go through the study information and procedures with you. If, after answering any questions you may have about the study, you are willing to participate, we will ask you to sign the consent form. After the consent form is signed, we will ask you a number of questions to enable us to enter you into the study and have your treatment allocated. We will also organise subsequent visits with you over the course of your pregnancy.

You will then be allocated by a computer-generated program to take either rifampicin (600mg daily in one or two doses) or UDCA (450-1000mg daily in single or divided doses, depending on the local protocol at [hospital]). You should take the study medication regularly in line with the instructions provided. You should continue with any other medications, including dietary supplements, that you are currently taking as advised. In general, there are no restrictions on other medications that might be necessary, although there are a few medications that have been shown to have a significant interaction with rifampicin. Therefore, it is important that you let us know should any of your medications change, especially if new ones are added. You should let your caring health professional know if you are taking rifampicin prior to any other medications being prescribed. It is also important that you advise us of any events that you suspect might be related to your treatment for ICP.

If you are taking UDCA, we will ask you, on the days when you need to have your blood tests, to delay taking the medication until after the morning blood sample has been drawn. This is to avoid measuring UDCA, itself a bile acid, in the blood test. Fasting is, however, not necessary for collection of blood to measure bile acids, whether you are taking rifampicin or UDCA.

**5 Other relevant information about the research project**

Severe early onset ICP is a rare disorder of pregnancy, which in Australia only affects one in every thousand pregnant women each year. We are hoping to recruit a total of just over 100 women to the study in ten Australian centres as well as university centres in London, Nottingham (both in the UK), Amsterdam (The Netherlands), Helsinki and Turku (Finland) and Gothenburg (Sweden), where we have collaborating researchers. We expect to recruit less than 10 women to the study at each site. Half of the women will be randomly allocated to take UDCA and half to rifampicin.

**6 Do I have to take part in this research project?**

Participation in any research project is voluntary. If you do not wish to take part, you do not have to. If you decide to take part and later change your mind, you are free to withdraw from the project at any stage.

If you do decide to take part, you will be given this Participant Information and Consent Form to sign and you will be given a copy to keep.

Your decision whether to take part or not to take part, or to take part and then withdraw, will not affect your routine treatment, your relationship with those treating you or your relationship with the [name] Hospital.

**7 What are the alternatives to participation?**

You do not have to take part in this research project to receive treatment at this hospital. Other options are available; these include the standard ursodeoxycholic acid (UDCA) and antihistamine medications, together with some other non-standard treatment drugs. Your study doctor will discuss these options with you before you decide whether or not to take part in this research project. You can also discuss the options with your local doctor.

**8 What are the possible benefits of taking part?**

We cannot guarantee or promise that you will receive any benefits from this research; however, possible benefits may include informing future patients of the advantages and disadvantages of the new treatment.

**9 What are the possible risks and disadvantages of taking part?**

Medical treatments often cause side effects. You may have none, some or all of the effects listed below, and they may be mild, moderate or severe. If you have any of these side effects, or are worried about them, talk with your study doctor. Your study doctor will also be looking out for side effects.

There may be side effects that the researchers do not expect or do not know about and that may be serious. Tell your study doctor immediately about any new or unusual symptoms that you get.

Many side effects go away shortly after treatment ends. However, sometimes side effects can be serious, long lasting or permanent. If a severe side effect or reaction occurs, your study doctor may need to stop your treatment. Your study doctor will discuss the best way of managing any side effects with you.

**Ursodeoxycholic Acid (UDCA)**

| Side Effect | How often is it likely to occur? | How severe might it be? | How long might it last? |
| --- | --- | --- | --- |
| Soft loose stools or diarrhoea | Between 1 in 10 and 1 in 100 women | Mild to moderate | While taking the medication |
| An itchy drug rash (hives) | About 1 in 10,000 women | Mild to moderate | While taking the medication |

**Rifampicin**

| Side Effect | How often is it likely to occur? | How severe might it be? | How long might it last? |
| --- | --- | --- | --- |
| Body fluids, including urine, stools, saliva, sputum, sweat and tears turn red-orange | Most women | Mild to moderate: not dangerous | While taking the medication |
| Headache, muscle pain, bone pain, heartburn, upset stomach, vomiting, stomach cramps, chills, diarrhoea | Less than 1 in 100 women | Mild to moderate: need to seek medical advice if persists | While taking the medication |
| Skin rash (hives), sores on skin or in the mouth, fever, yellowing of the skin or eyes | Between 1 in 100 and 1 in 1000 women | Serious: seek urgent medical advice | While taking the medication |

NB If you are not sure about possible changes in your skin or eye colour, please discuss with your health practitioner.

Please note that it has been reported that soft contact lenses may be permanently stained by rifampicin. Therefore, if you have soft contact lenses, you may wish to consider carefully the implications of being allocated to rifampicin treatment.

If, during this study, you are found to have a medical condition of which you and your healthcare personnel were previously unaware, appropriate referral can be made to medical/genetic specialists for further advice within the [location] Health system. It is possible, though unlikely, that the discovery of such a condition *might* affect your health insurance in the future.

Any side effects from treatment will be managed within your [local public health] system.

No negative effects of rifampicin on the unborn child and on the newborn baby have been reported with its use in treatment of pregnant women with tuberculosis. It is, however, recommended that, as per standard practice, all babies should receive an injection of vitamin K soon after birth, in particular because of a theoretical impact of rifampicin on vitamin K metabolism, leading to an increased risk of bleeding in the newborn. If you do not wish for your baby to receive this standard therapy, you will not be permitted to take part in the study.

If you become upset or distressed as a result of your participation in the research, the study doctor will be able to arrange for counselling or other appropriate support. Any counselling or support will be provided by qualified staff who are not members of the research project team. This counselling will be provided free of charge.

Having a blood sample taken may cause some discomfort, bruising, minor infection or bleeding. If this happens, it can be easily treated.

**10 What will happen to my test samples?**

Routine blood and urine samples will be collected for any testing your doctor might order to monitor your health. We would also like to collect some of the placenta for routine examination when your baby is born.

**11 What if new information arises during this research project?**

Sometimes during the course of a research project, new information becomes available about the treatment that is being studied. If this happens, your study doctor will tell you about it and discuss with you whether you want to continue in the research project. If you decide to withdraw, your study doctor will make arrangements for your regular health care to continue. If you decide to continue in the research project, you will be asked to sign an updated consent form.

Also, on receiving new information, your study doctor might consider it to be in your best interests to withdraw you from the research project. If this happens, he/ she will explain the reasons and arrange for your regular health care to continue.

**12 Can I have other treatments during this research project?**

Whilst you are participating in this research project, you may not be able to take some or all of the medications or treatments you have been taking for your condition or for other reasons. It is important to tell your study doctor and the study staff about any treatments or medications you may be taking, including over-the-counter medications, vitamins or herbal remedies, acupuncture or other alternative treatments. You should also tell your study doctor about any changes to these during your participation in the research project. Your study doctor should also explain to you which treatments or medications need to be stopped for the time you are involved in the research project.

There are a few medications that have significant interaction with rifampicin, although almost all of these would not be commonly prescribed during pregnancy. Your doctor or research assistant will have a list of such medications and will check whether any medication that you are taking will mean that you cannot be recruited into the study. If you are commenced on such medications during the time of the study, and you are taking rifampicin, we will have to withdraw you from the study, although we would still wish to collect information about the outcome of the pregnancy, with your permission.

It may also be necessary for you to take medication during or after the research project to address side effects or symptoms that you may have. You may need to pay for these medications and so it is important that you ask your doctor about this possibility.

**13 What if I withdraw from this research project?**

If you decide to withdraw from the project, please notify a member of the research team before you withdraw. This notice will allow that person or the research supervisor to discuss any health risks or special requirements linked to withdrawing.

If you do withdraw your consent during the research project, the study doctor and relevant study staff will not collect additional personal information from you, although personal information already collected will be retained to ensure that the results of the research project can be measured properly and to comply with law. You should be aware that data collected by the sponsor up to the time you withdraw will form part of the research project results. If you do not want them to do this, you must tell them before you join the research project.

**14 Could this research project be stopped unexpectedly?**

This research project may be stopped unexpectedly for a variety of reasons. These may include reasons such as:

- Unacceptable side effects from the drug/treatment
- The drug/treatment being shown not to be effective
- The drug/treatment being shown to work and not need further testing.

**Part 2 How is the research project being conducted?**

**15 What will happen to information about me?**

By signing the consent form, you consent to the study doctor and relevant research staff collecting and using personal information about you for the research project. Any information obtained in connection with this research project that can identify you will remain confidential. Identifiable information will be held in a secure, password protected database at the site where you join the study. Any paper forms with your name and other identifiable details will be stored in locked filing cabinets on the site. Re-identifiable information collected for the study, “study data” will be entered and stored on a password protected research database. Only research personnel at this site will have access to your data. Your information will be used for the purpose of this research project and it will only be disclosed with your permission, except as required by law.

Information about you may be obtained from your health records held at this and other health services for the purpose of this research. By signing the consent form, you agree to the study team accessing health records if they are relevant to your participation in this research project.

As severe early onset ICP is a very rare condition, any information we have collected about you, on the treatment you receive, and the results of the pregnancy and birth, is very valuable, in a research sense. Therefore, with your permission, we would like to keep de-identified information about you and this pregnancy in a database. This would be used as a research resource and “pooled” with information about women with ICP in the future. The information will be stored in a password protected database, on a secure network at The University of Adelaide. Any future uses of this information would need to be approved by a certified Human Research Ethics Committee.

Your health records and any information obtained during the research project are subject to inspection (for the purpose of verifying the procedures and the data) by the relevant authorities and authorised representatives of the Sponsor, The University of Adelaide, the institution relevant to this Participant Information Sheet, *[name of]* Hospital, or as required by law. By signing the Consent Form, you authorise release of, or access to, this confidential information to the relevant study personnel and regulatory authorities as noted above.

It is anticipated that the results of this research project will be published and/or presented in a variety of forums. In any publication and/or presentation, information will be provided in such a way that you cannot be identified, except with your permission.

Information about your participation in this research project may be recorded in your health records.

In accordance with relevant Australian and/or *[enter State]* privacy and other relevant laws, you have the right to request access to your information collected and stored by the research team. You also have the right to request that any information with which you disagree be corrected. Please contact the study team member named at the end of this document if you would like to access your information.

Any information obtained for the purpose of this research project that can identify you will be treated as confidential and securely stored. It will be disclosed only with your permission, or as required by law.

We will retain the data that we have collected about you and your baby for [enter number] years as required by [State of Australia] [organisation name: eg Department of Health] data retention policies.

**16 Complaints and compensation**

If you suffer any injuries or complications as a result of this research project, you should contact the study team as soon as possible and you will be assisted with arranging appropriate medical treatment. If you are eligible for Medicare, you can receive any medical treatment required to treat the injury or complication, free of charge, as a public patient in any Australian public hospital.

In the event of loss or injury, the parties involved in this research project have agreed to abide by the standard Medicines Australia Indemnity and Compensation Guidelines (https://medicinesaustralia.com.au/policy/clinical-trials/indemnity-and-compensation-guidelines/)

**17 Who is organising and funding the research?**

This research has been initiated by the senior study doctor, Professor Bill Hague, and has been funded by the Medical Research Futures Fund (MRFF) of the Australian government. The study is being coordinated by the Robinson Research Institute at the University of Adelaide, the Australian sponsor of the study. The study is being conducted at several hospitals in Australia, the United Kingdom, the Netherlands, Finland and Sweden.

The study is being conducted at [hospital name] by [investigator name].

You will not benefit financially from your involvement in this research project.

In addition, if knowledge acquired through this research leads to discoveries that are of commercial value to The University of Adelaide, the study doctors or their institutions, there will be no financial benefit to you or your family from these discoveries.

No member of the research team will receive a personal financial benefit from your involvement in this research project (other than their ordinary wages).

**18 Who has reviewed the research project?**

All research in Australia involving humans is reviewed by an independent group of people called a Human Research Ethics Committee (HREC). The ethical aspects of this research project have been approved by the HREC of the Women’s and Children’s Health Network. Approval for the project has been given by both The University of Adelaide as sponsor and the [name of local] Hospital.

This project will be carried out according to the *National Statement on Ethical Conduct in Human Research (2007)*. This statement has been developed to protect the interests of people who agree to participate in human research studies.

**19 Further information and who to contact**

The person you may need to contact will depend on the nature of your query.

If you want any further information concerning this project or if you have any medical problems which may be related to your involvement in the project (for example, any side effects), you can contact the principal study doctor on +61 (0)8 8161 7000 or the study coordinator: Dr Suzette Coat on +61 (0)401 055 150 or +61 (0)8 8313 1338.

**Clinical contact person**

| Name | *PI Name* |
| --- | --- |
| Position | *[position]* |
| Telephone | *[Phone number]* |
| Email | *[Email address]* |

For matters relating to research at the site at which you are participating, the details of the local site complaints person are:

**Complaints contact person**

| Name | *[Name]* |
| --- | --- |
| Position | *[Position]* |
| Telephone | *[Phone number]* |
| Email | *[Email address]* |

If you have any complaints about any aspect of the project and the way it is being conducted, or any questions about being a research participant in general, then you may contact:

**Reviewing HREC approving this research** **and HREC Executive Officer details**

| Reviewing HREC name | *Women’s and Children’s Hospital Network HREC* |
| --- | --- |
| HREC Executive Officer | *Luke Fraser* |
| Telephone | *+61 (0)8 8161 6521* |
| Email | *luke.fraser2@sa.gov.au* |

**Consent Form**

| **Title** | *A randomised Trial of URsodeoxycholic acid versus RIFampicin in severe early-onset Intrahepatic Cholestasis of pregnancy: the TURRIFIC study.* |
| --- | --- |
| **Short Title** | *TURRIFIC* |
| **Lay Title** | *A Trial comparing URsodeoxycholic acid with RIFampicin in severe early onset Intrahepatic Cholestasis of pregnancy: the TURRIFIC study* |
| **Project Sponsor** | *The University of Adelaide* |
| **Principal Investigator** |  |
| **Location** |  |

**Declaration by Participant**

***CONSENT FORM***

***I ______________________________________________________________________***

hereby consent to my involvement in the research project entitled:

***A randomised Trial of URsodeoxycholic acid versus RIFampicin in severe early-onset Intrahepatic Cholestasis of pregnancy: the TURRIFIC study.***

1. I understand the nature and purpose of the research project described on the attached Information Sheet and agree to taking part.

2. I understand that I may not directly benefit by taking part in this study.

3. I acknowledge that the possible risks and/or side effects, discomforts and inconveniences, as outlined in the Information Sheet, have been detailed in the attached Information Sheet.

4. I understand that I can withdraw from the study at any stage and that this will not affect medical care or any other aspects of my relationship with my doctor or this healthcare service.

5. I understand that there will be no payment to me for taking part in this study.

6. I have had the opportunity to discuss taking part in this research project with a family member or friend.

7. I am aware that I should retain a copy of the Consent Form, when completed, and the Information Sheet.

8. I agree to the accessing of my medical records and of my baby for the purpose of this study.

9. I understand that my information will be kept confidential as explained in the information sheet except where there is a requirement by law for it to be divulged.

10.Specific consents:

1. I agree to the collection of my placenta for laboratory examination, for a de-identified copy of the pathology report and de-identified microscopic sections to be sent for central review in Adelaide and for its subsequent storage in a dedicated biobank.

□ YES □ NO

1. I consent to be contacted after this pregnancy to be invited into follow up studies about myself and my child.

□ YES □ NO

1. I consent to non-identifiable data being used for future research and childhood development research that is approved by a certified Human Research Ethics Committee.

□ YES □ NO

Signed: .........................................................

Full name of participant: ......................................................................................

Dated:.............................

*I certify that I have explained the study to the participant and consider that she understands what is involved.*

*Signed: .................................................... Title: .......................................................*

*Dated: ...............................*
